# Upcycling of regular wood trunks and logs using wave function collapse (WFC), augmented reality (AR), and mixed reality (MR) technologies for circular design

**DOI:** 10.1038/s41598-025-20398-8

**Published:** 2025-10-21

**Authors:** Jianing Luo, Boyuan Yu, Yuan Jiang, Adam Fingrut, Adam Holloway

**Affiliations:** 1 Oxford School of Architecture, Oxford Brookes University, Headington, United Kingdom; 2 School of Architecture, The Chinese University of Hong Kong, Hong Kong, Hong Kong; 3 School of Architecture and Design, Beijing Jiaotong University, Beijing, China; 4 The Bartlet School of Architecture, University College London, London, United Kingdom

**Keywords:** Upcycling wood, Plastic bottles, Repurposed heat-moldable joinery, Wave function collapse (WFC), Augmented reality (AR), Mixed reality (MR), Engineering, Civil engineering, Environmental impact, Sustainability

## Abstract

In sustainable building design and construction (SBDC), irregular timber elements, such as unprocessed logs, forks, and branches, remain significantly underutilised due to their complex geometries, which complicate reconfiguration and lead to considerable material waste. This study addresses this challenge by introducing a computational workflow that optimises the upcycling of irregular wood into feasible building components. The CAAD contribution of this work lies in applying Wave Function Collapse (WFC) as a digital aggregation method, enabling automated spatial configuration of irregular wood elements. This method integrates 3D scanning, algorithmic aggregation, and finite element analysis (FEA) to assess structural viability, ensuring efficient material utilisation. For joinery experiments, our engineering application involves the development of a heat-moldable joinery method using recycled PET bottles, which eliminates the need for adhesives or mechanical fasteners. By leveraging the heat-shrink properties of PET, structurally stable connections are formed between upcycled wood components. The proposed framework is demonstrated through the fabrication of functional furniture and pavilion-scale architectural prototypes, showcasing an innovative approach to material repurposing. This study advances Computer-Aided Architectural Design (CAAD) and construction techniques by 3D scanning, volumetric design, AI-driven building scale ideation and Augmented Reality (AR) and MR-assisted assembly, demonstrating scalable solutions for sustainable architecture and circular material reuse.

**Supplementary Information:**

The online version contains supplementary material available at 10.1038/s41598-025-20398-8.

## Introduction

The interconnectedness between forests and climate change is well-documented^1^. Climate plays a critical role in determining forest distribution, composition, and health, while the exploitation of forests, in turn, influences broader climate dynamics^2,3^. Over-exploitation of forests has exacerbated global warming and environmental changes. In response, forestry agencies have advocated for sustainable practices to enhance ecological construction and protection, aiming to balance resource use with environmental preservation^4^. Sustainability, closely linked to the responsible use of renewable resources, emphasises avoiding over-extraction that leads to excessive resource strain^5^. On the other hand, they are fully utilised to encourage efficient collection of wood produced in the “from forest to sawmill supply chain” to achieve sustainable forest resources^6^.

Wood is a crucial renewable resource, widely used in energy production, construction, furniture, textiles, and paper. However, its increasing demand, long growth cycle, and economic and environmental constraints make it a limited resource^7,8^. Projections indicate that wood production will fall short of societal needs by 2030^9^. Therefore, efficient wood resource utilization and waste wood management are essential, necessitating collection, recycling, and reuse of idle wood^10–12^. In developed countries, waste wood recycling accounts for only 3.1 million tons, about 17% of total waste wood^13^. Research on wood recycling and processing plants has found that wood recycling does not inherently prevent wood from becoming waste but simply delays final disposal. Therefore, it highlights the need to reset wood lifecycle through innovative design to maximize recycling rates^14^. This approach aims to reduce waste storage in landfills and wood mills, promoting the development of viable recycling and disposal plans.

The Circular Economy (CE) paradigm promotes upcycling waste products, the process of transforming waste materials or unwanted products into new materials or products of higher quality and greater environmental value, aligning with consumer responsibility and environmental demands, fostering an industry focused on value-added production^15^. Construction activities significantly strain natural resources and generate substantial waste, impeding sustainable development^16^. Effective construction demolition waste recycling involves downgrading, recycling, and reuse, often through processing into aggregates for new structures. However, the construction industry, based on discrete construction principles, considering the original components and the characteristics of being reassembled and used has launched a lot of attempts on the original non-degraded recycling and reuse methods^17^. It is also regarded as an effective way to alleviate wood consumption and realize the reset life cycle of wood. The assembly and design of original parts, dismantling methods, connection methods, and standardized production automation manufacturing have become an important part of the discussion. Furthermore, strategies for completing deconstruction and reconfiguration into new structures in later stages are key topics. Crucially, standardizing these processes—design, dismantling, connections, automation, and reconfiguration—enables even unskilled workers to master these techniques quickly.

Architectural precedents offer key insights into material reuse and innovative technologies^18^. Simple upcycled furniture—such as stools and tables made from logs or stumps—demonstrates the potential of waste wood but is often limited by its low structural demands and simple fabrication methods^19^. These examples, frequently seen in exhibitions and online platforms, rely on basic operations such as cutting, gluing, and sanding. More complex architectural applications, such as pavilions, require robust, structurally sound geometries and precise assembly techniques. The irregularity of reclaimed logs demands careful material pairing and structurally viable joints. The Kernen Stetten playground^20^, constructed from forked branches and irregular logs, exemplifies these challenges. Its unique layout, combining vertical and horizontal log assemblies, illustrates the limitations of current workflows, especially in terms of replicability and disassembly. Glue and nails, commonly used for such structures, hinder future reuse.

To overcome these limitations, advanced technologies-including Computer-Aided Architectural Design (CAAD), robotics, and Augmented Reality (AR)-assisted assembly-are increasingly integrated into wood upcycling workflows. Projects such as the AA School’s Wood Chip Barn^21^ and Timber De-Standardised^22^ have demonstrated the viability of CAAD-driven workflows. However, broader application and integration of AR and robotics are needed to enhance scalability, accuracy, and efficiency in construction.

This article explores the potential of upcycling reclaimed, easy-to-reconfigure wood—such as tree trunks, forks, and logs-into functional and innovative furniture, installations, and architectural structures. The central hypothesis is that integrating digital modelling and Mixed Reality (MR) that can significantly improve construction precision, reduce labour intensity, and scale up sustainable practices. This framework links material databases, geometric simulation, and multi-scalar physical prototyping to explore how reclaimed wood can support circular construction practices. Meanwhile, a heat-moldable joinery method using repurposed PET plastic bottles is introduced to the workflow, offering a lightweight, adaptable, and low-cost solution for connecting irregular timber elements with minimal tooling requirements. The proposed Sustainable Building Design and Construction (SBDC) framework addresses this by connecting digital and physical processes through material databases, geometric simulations, and multi-scalar construction experiments. The framework aims to develop scalable, aesthetic, and functional workflows that support the broader integration of circular wood reuse into urban environments.

This research investigates how upcycled wood can be effectively utilised to create furniture, pavilions, installations, and architectural elements, with a focus on both design innovation and sustainable construction practices. It aims to identify the key workflows and strategies necessary for efficient wood upcycling, establishing robust and repeatable methods that support circularity in the built environment. In doing so, the study explores the integration of advanced technologies into the design and fabrication process to enhance precision, reduce labour intensity, and increase scalability. Through this approach, the research seeks to develop functional and aesthetically compelling artefacts using reclaimed materials, while proposing viable solutions for embedding these practices within urban and architectural contexts.

## Literature review

### Linear vs. volumetric: Reuse and repurposing irregular Wood-based circular Building design and construction using CAAD

**Fig. 1:**
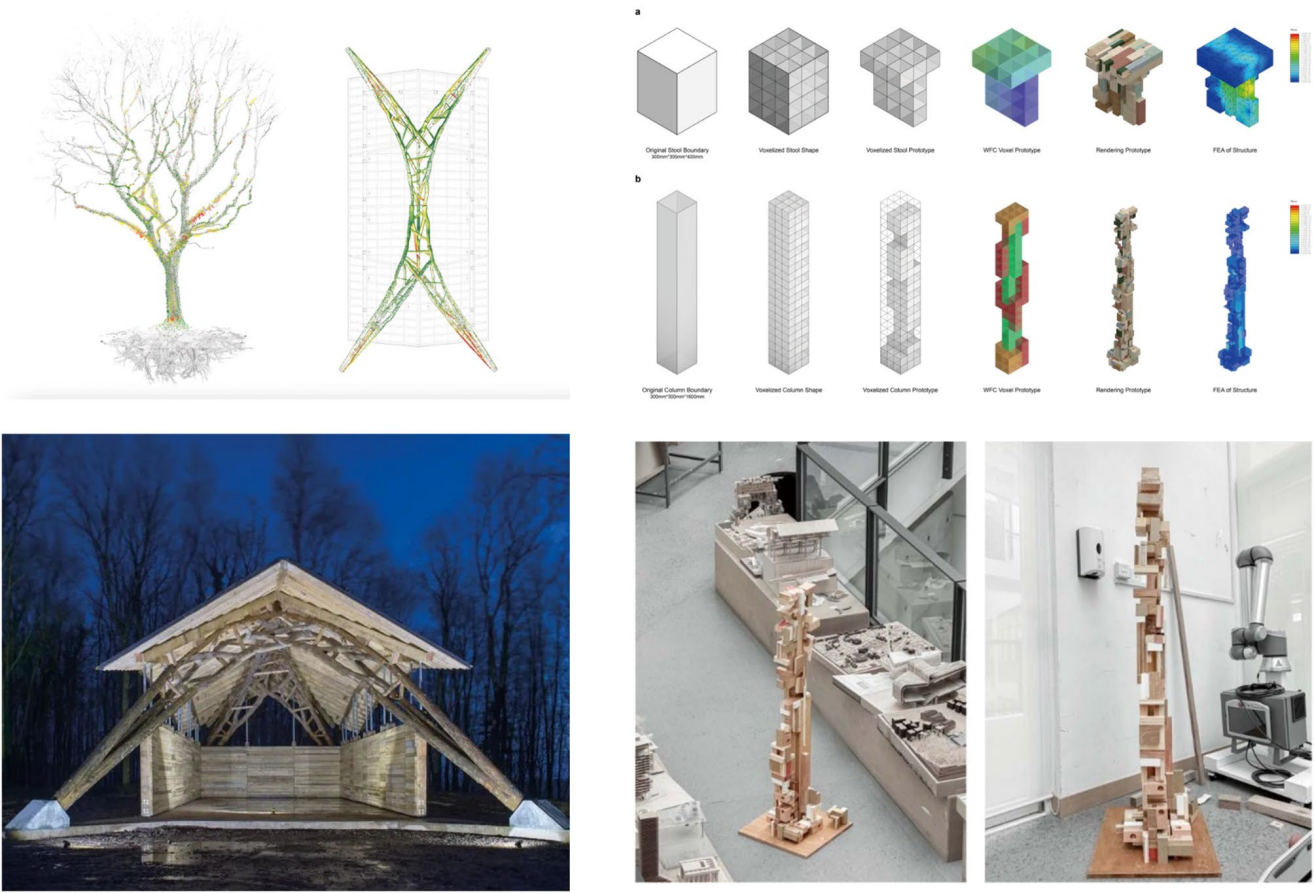
Wood Chip Barn^21^ vs. Volumetric-based circular building design^23^.

The reuse and repurposing of wood in architecture has gained increasing traction as the construction industry seeks circular, low-carbon alternatives to conventional material sourcing. CAAD methods have enabled architects to engage more intelligently with irregular wood, including off-cuts, branches, and forks, by treating them not as waste but as uniquely shaped design resources.

Traditional wood construction relies on predictable, standardised material geometries; however, recent research explores how irregular wood can serve as meaningful architectural components. A landmark experimental case is the robotically fabricated barn by Architectural Association students in Dorset, UK^24^ (Fig. 1). This project exemplifies a “material-first” approach where naturally forked and branched wood elements are not trimmed to fit conventional templates but instead celebrated as structural units. The design team used CAAD and robotic fabrication to map, sort, and strategically deploy these irregular components according to their inherent geometric and mechanical properties. By treating line and length not as constraints but as intrinsic characteristics of the component, the project redefines assembly as a bottom-up process that responds to material variation. While pioneering, this approach relied on bespoke robotic fabrication, limiting its accessibility and scalability for wider use. Our work seeks to democratise the fabrication process by developing a workflow that leverages more accessible MR-assisted manual assembly, empowering non-experts to work with similar geometric complexity.

While such work offers aesthetic and conceptual innovation, the challenge lies in scaling and systematising these workflows. The integration of generative algorithms, such as voxel-based design methods, has enabled new ways to handle large datasets of non-standard material units. Also, in a previous study we introduced a voxel-based system that uses reclaimed off-cut wood aggregated into modular volumes^25^ (Fig. 1). Each voxel serves as both a design unit and a structural component, whose properties are informed by 3D scanning and evaluated through finite element analysis (FEA). Our study extends this volumetric approach by integrating a modified WFC algorithm not just for aggregation, but for generating functionally distinct substructures (e.g., seats vs. legs). Crucially, we close the design loop by directly linking this generative voxel data to an FEA validation stage, enabling rapid, performance-informed iteration and ensuring structural viability from the earliest design stages.

The volumetric voxel model and the forked-branch assembly share conceptual similarities. Both operate on the logic of discrete units, whether geometric (voxels) or organic (branches), and rely on CAAD-driven systems to organise complexity. Crucially, both approaches move away from standardisation and instead embrace heterogeneity as a design opportunity. These strategies align with broader efforts in circular architecture to match available materials to emerging needs, thereby minimising waste and maximising design potential.

### Integrating AI, augmented reality based - decision making (AR-DM) for scalable, inclusive, and circular architectural design

In the transition toward sustainable and circular design paradigms, the integration of Artificial Intelligence (AI), Augmented Reality (AR), and Mixed Reality (MR) presents transformative opportunities for the Architecture, Engineering, and Construction (AEC) industry. These technologies reshape design workflows—from ideation through construction—supporting scalability, participatory engagement, and effective reuse of reclaimed materials^26^.

### Augmented reality (AR) and mixed reality (MR)

AR and MR generally represent distinct dimensions on the reality-virtuality continuum^27^. AR is primarily characterized by the overlaying of digital information—such as text, images, or 3D models—onto the user’s view of the physical world, typically via smartphones or tablets^28^. The interaction between digital and physical elements is often limited; AR enhances visualization but does not typically create a spatially aware, interactive environment where digital content can be occluded by real objects. MR represents where virtual objects are not merely overlaid but are integrated into and can interact with the real environment^29^. Enabled by sophisticated hardware like the Microsoft HoloLens, MR systems create spatially anchored holograms that can be manipulated by the user and can be occluded by real-world objects. In this study, we use AR for scalable design visualisation and participatory decision-making (as seen in the pavilion proposal) and MR for hands-on, interactive assembly guidance (as applied in the furniture fabrication).

### AI in computational and circular design

AI-assisted design tools have been embedded in early-stage architectural workflows, enabling material-informed exploration and decision-making^30,31^. Generative AI, a branch of machine learning, excels at navigating extensive material databases, performing structural assessments, and matching resources to design requirements. For example, Önalan et al.^32^ demonstrated how generative approaches enhance form-finding and resource alignment for informal or irregular components. Additionally, AI systems are now capable of scanning salvaged wood inventories and automatically suggesting applications, while optimizing structural layouts and preempting fabrication conflicts^33,34^.

### AR & MR in participatory design and assembly

AR technologies have been shown to boost productivity, stakeholder engagement, and assembly accuracy in reclaimed-wood construction projects. A case in point is the creation of a double-curved shell structure using irregular timber, where AR guided wood nailing and assembly sequencing^35^. AR also enables immersive design review, real-time prototyping, and error reduction through contextual overlays^36^. MR, on the other hand, supports democratized fabrication by guiding non-expert users through step-by-step assembly tasks using mixed-reality instructions, enhancing precision and reducing errors in circular timber workflows^37^.

While existing research emphasizes material-driven digital workflows and physical prototyping, AR and MR technologies are positioned here as strategic future directions. Their adoption holds promise for addressing the challenges of processing irregular reclaimed materials and achieving inclusive, precise, and scalable circular construction.

## Methods and materials

This research proposes a sustainable approach to the design and construction of buildings using upcycled trunks and logs. It integrates advanced technologies, including 3D scanning, FEA, and MR-assisted assembly workflows to support this.

### Material recording and assessment

A novel digital retrieval workflow forms the core of the material assessment methodology. This workflow employs 3D scanning as the primary method to capture the precise geometry and condition of salvaged wood components. enabling accurate material characterization and structural potential classification. For example, Pelczynski et al.^38^ demonstrated how LIDAR and photogrammetry workflows can support mechanical property prediction and classification of reclaimed timber beams. Tamke et al.^39^ demonstrated a CT‑scan‑based non‑destructive pipeline capable of assessing geometry, density distribution, and mechanical integrity in reclaimed timber elements. Based on the scanned data, each component is classified into a Material Hierarchy for Structural Potential^40^, defined as follows:

The classification includes five categories, reflecting a continuum of perceived structural viability:

Grade A - No structural potential: The components are highly degraded, showing extensive cracks, dead knots, and voids.

Grade B - Short grain: These components contain holes, knots, and must be recomposed into panels or other consolidated forms to achieve structural functionality.

Grade C - Medium grain length with low degradation: Similar to Grade B, these components must be reassembled into panels or composite elements to serve structurally.

Grade D - Long and continuous grain structure with low degradation: These components can be made structurally viable through minimal recovery processes.

Grade E - Long and continuous grain structure with no visible degradation: These elements are fully suitable for structural applications without the need for extensive modification.

### Voxelization and statistics of irregular wood

**Fig. 2:**
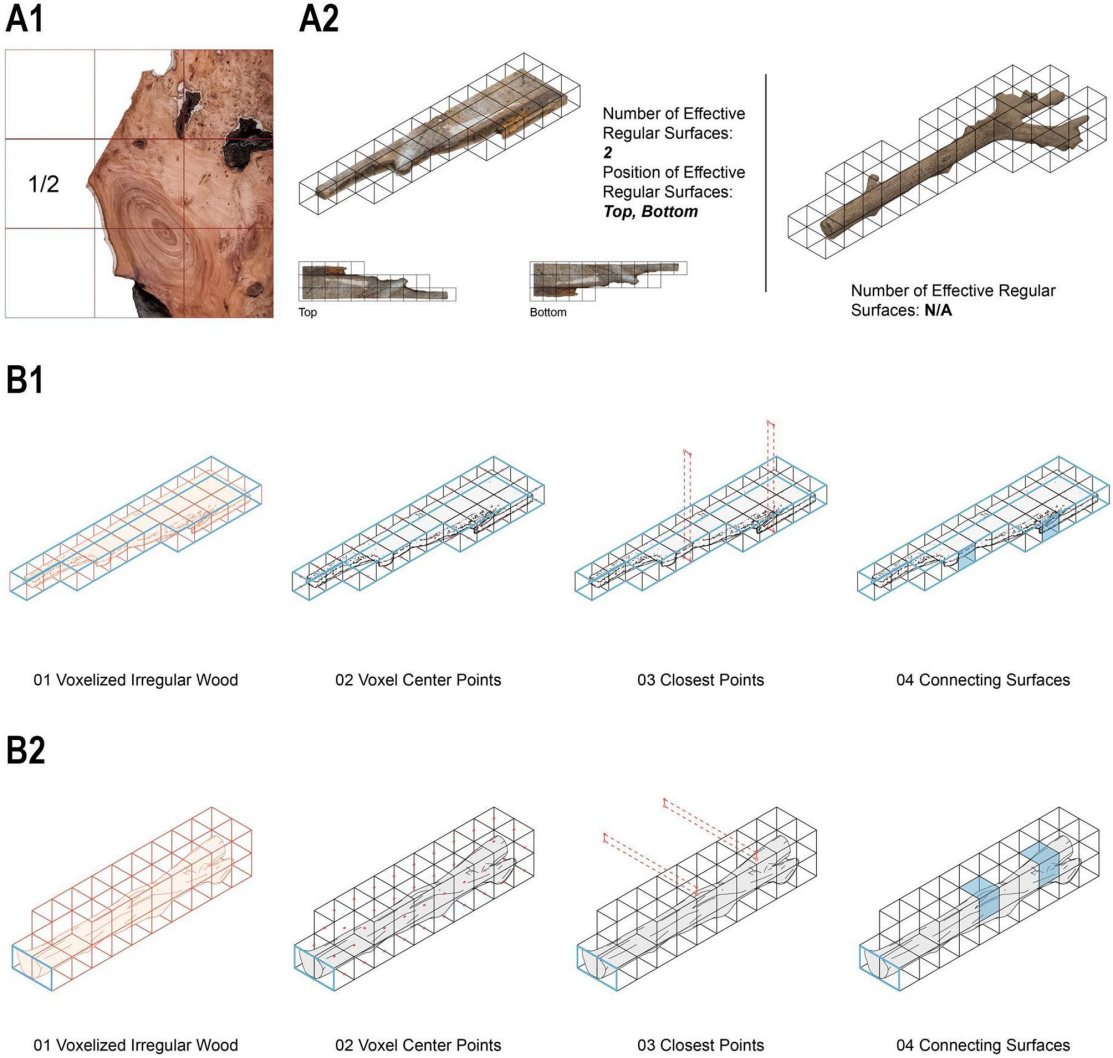
Voxelization and statistics of irregular wood and connection rules for each voxel and fragments.

Voxelization is a process that converts a 3D geometric model into a set of discrete, volumetric units called voxels (volumetric pixels), analogous to how a 2D image is represented by pixels. This technique simplifies complex, irregular shapes into a structured 3D grid, making them computationally manageable for analysis and generative design. This methodical approach to voxel-based packing is the first step when designing with irregular materials such as reclaimed wood for structural applications. The digital model divides the wood into a three-dimensional grid of uniform cubes (voxels). This 50% threshold was selected after a comparative assessment of various filling patterns, total voxel counts, and internal fill ratios, demonstrating optimal results in terms of balancing geometric approximation with material representation for irregular shapes. (Fig. 2A). This selected filling rate is highly related to voxel size selection. Firstly, we voxelize the voxel following 40 mm, 50 mm,60 mm,70 mm,

According to the existing materials, 50 is gradually used as the best unit for digital packing of later materials. When 50 mm is used as the basic unit, we choose to ignore the materials below 50%. This part is also considered within the tolerance rate of processing loss, so we choose the threshold of 50%. For materials above 50, we choose to use off-cut materials during processing.

Surface detection for Connection - Each voxel is classified based on its position and the characteristics of the material it represents. The classification identifies ‘effective regular surfaces,’ which are flat and regular enough to serve as connection points. In the given example, these surfaces are categorized as ‘Top’ and ‘Bottom’ based on their orientation and usability for structural connections.

Material Rationalization and Replacement - The next step is rationalising material usage once the voxel grid and connectivity rules are defined. This involves optimizing the arrangement of voxels to minimize waste and ensure efficient use of irregular materials. Irregular shapes are often replaced with more regular voxel-based representations to simplify the assembly process while retaining the material’s unique aesthetic and structural properties.

### Structure assessment

FEA is a powerful computational method used to predict how an object will react to real-world forces. By breaking down a complex object into many small, simple elements, FEA can simulate stress distribution, deformation, and overall stability, providing critical insights into how the material will behave in real-world applications. Töpler and Buchholz^41^ established guidelines for finite element–based design of timber structures, demonstrating how FEA can reliably assess stress, strain, and connection behaviors to support informed decision-making in timber construction workflows. Users can adjust the design based on performance data, optimizing the structure for maximum efficiency and safety. By iterating through different configurations and loads, FEA helps identify the most effective use of each piece of wood and contributes to the integrity and functionality of the final product. This process significantly reduces material waste, which predicts and improves performance before physical assembly.

### Fabrication: MR-assisted assembly method

The integration of Mixed Reality (MR) technology, specifically utilising Microsoft HoloLens and Fologram, plays a pivotal role in facilitating the assembly of upcycled irregular timber, making the fabrication work more accessible, even for non-professionals. This method commences with the precise 3D scanning of irregular timber pieces to generate detailed digital models.

During the assembly phase, the HoloLens headset overlays these digital models directly onto the physical workspace, while Fologram provides real-time interaction and step-by-step guidance. Users wearing the HoloLens headset can visualise the digital design precisely in their field of vision, allowing them to follow Fologram’s instructions for accurate alignment and fitting of each irregular timber piece.1 This immersive approach significantly reduces the inherent complexity associated with handling timber pieces of non-standard shapes and sizes, thereby optimising material use and contributing to the overall structural integrity of the assembly.

While this study demonstrates the potential of MR for intuitive guidance and visualisation in the assembly of irregular timber, it is important to contextualise claims regarding enhanced precision, error reduction, and safety. Mixed Reality technologies can improve construction precision, reduce errors, and enhance safety, as evidenced by other research in AR-assisted timber fastening which has shown sub-millimetre accuracy in positioning tasks. However, in the context of this specific study, the primary focus of MR integration is on providing accessible and intuitive guidance for non-expert users, facilitating the assembly process of complex, non-standard components. Empirical validation of construction-level precision achieved by unskilled participants using this specific MR setup would require dedicated user studies and quantitative performance metrics, which are beyond the scope of the current demonstration and are areas for future research. The current application focuses on visual guidance and interactive feedback that empowers users to accurately position and fit irregular components, thereby lowering the barrier to participation in sustainable construction practices.

**Fig. 3:**
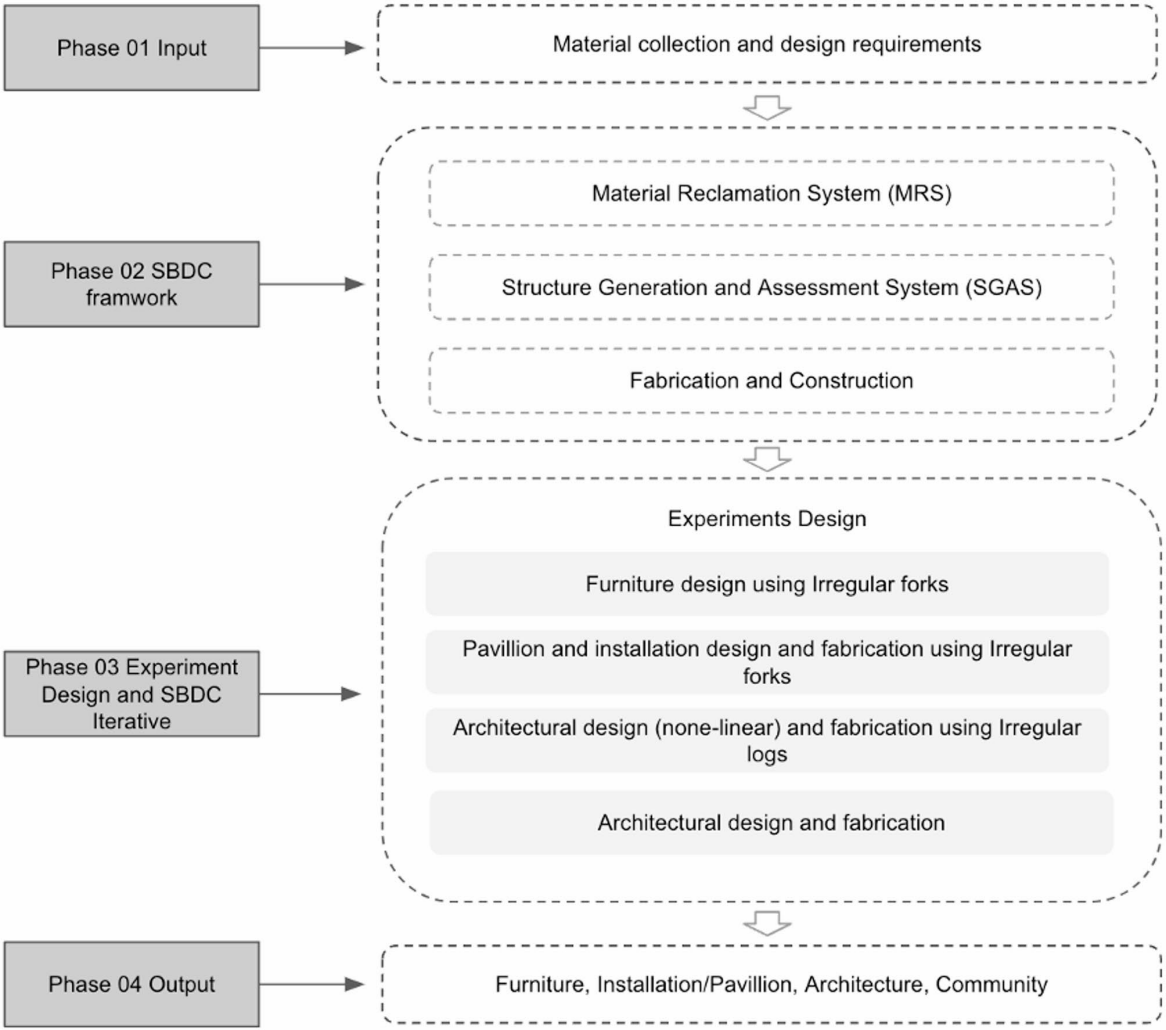
Research design.

This research design plan follows a structured four-phase approach to explore the potential of upcycling irregular wood in sustainable building design and construction (Fig. 3). Phase 01 involves the input stage, focusing on material collection and defining design requirements. This sets the foundation for Phase 02, which implements the SBDC framework comprising three critical systems: Material Reclamation System (MRS), Structure Generation and Assessment System (SGAS), and Fabrication and Construction. The MRS ensures systematic collection and classification of irregular wood, while the SGAS integrates advanced structural design and evaluation technologies. Phase 03 encompasses experiment design and iterative SBDC processes, categorizing experiments into four distinct areas: furniture design using irregular forks, pavilion, installation design and fabrication using irregular forks, architectural design (non-linear) and fabrication using irregular logs, and general architectural design and fabrication. Each experiment addresses unique design challenges and potential applications, emphasizing the iterative nature of the process where feedback from each experiment informs subsequent phases, refining techniques and outcomes. Finally, Phase 04 outputs the results, showcasing furniture, installations, architectural elements, and community applications. This hierarchical, multi-scale, and feedback-driven approach ensures comprehensive exploration and optimization of upcycling practices and tests our framework’s repeatability and research feasibility.

## Results

### SBDC system: framework of irregular wood-based design and construction

**Fig. 4:**
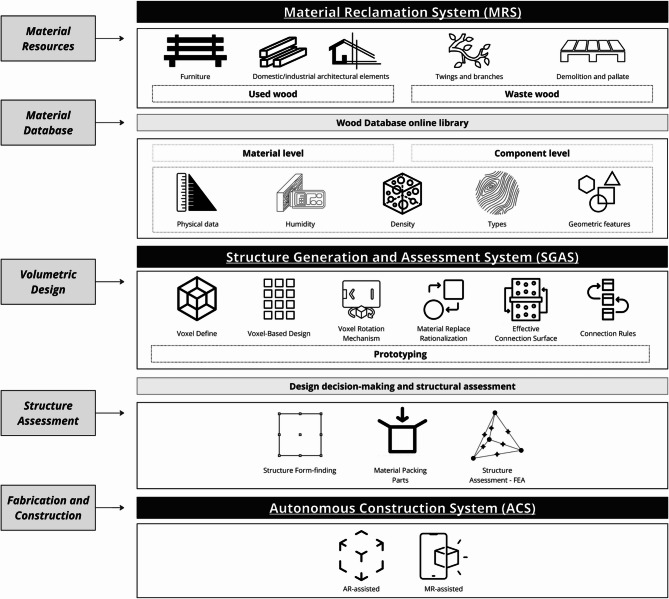
Sustainable building design and construction (SBDC).

The proposed study explores the SBDC framework for upcycling irregular wood into building and furniture elements. This framework integrates advanced technologies such as Machine Learning, 3D scanning, FEA, and MR-assisted assembly. The process is divided into three main systems: Material Reclamation System (MRS), which handles the collection and digital cataloging of reclaimed wood; Structure Generation and Assessment System (SGAS), which uses algorithms to generate and structurally validate designs; and Fabrication. The detailed workflow is outlined below, following the provided diagram (Fig. 3).

### Material reclamation system (MRS)

Step 1: Material Resource Collection - The first phase involves collecting various types of wood, including used wood from furniture and architectural elements, waste wood from demolition and pallets, twigs and branches, and off-cut wood from sawmills and factories. Step 2: Data Collection and Classification - Geometrical Features: The geometrical features of the collected wood are recorded using 3D scanning technologies. Large-scale pieces are scanned using drones like the DJI Air 2 S, mesoscale pieces with Reality Capture technology using Sony A6400 cameras, and small-scale pieces using handheld 3D scanners like EinScan-SE and EinScan Pro 2X. Physical Characteristics: Physical parameters such as moisture content are measured using devices like the R&D-MT-26 Moisture Meter and the BENETECH-GM630 wood moisture meter. Step 3: Data Collation and Classification - The collected data is organized into a comprehensive material database where each wood element is assigned a unique identifier. The material is classified based on its key attributes to create a searchable inventory. The primary classification categories are: Geometrical Profile: Includes dimensions, volume, and overall shape derived from the 3D scan data; Physical Properties: Categorizes the wood by species, moisture content percentage, and density; Quality Grade: A grade is assigned based on the structural condition (five categories). This system ensures a structured input for subsequent stages.

### Structure generation and assessment system (SGAS)

Step 4: Voxel Definition and Input Matrix - The process begins by converting each unique wood element from the material database into a discrete block of voxels, creating a digital twin of the physical component. The voxel size is defined according to the scale and geometric detail of the original wood piece. This collection of distinct voxel blocks forms the digital inventory, or input matrix, for the structure generation phase. Step 5: Structure Generation - A generative algorithm assembles a larger structure by selecting and arranging these voxel blocks from the inventory. During this process, a rotation mechanism is applied to find the optimal placement for each component. To be clear, this mechanism operates on entire wood elements, rotating each complete block of voxels as a single, rigid unit; it does not rotate individual voxels. The system tests various orientations for each element to satisfy a set of connection rules, such as maximising surface contact or aligning grain directions, thereby ensuring robust links and structural integrity. Step 6: Chair Typology Voxelization and FEA - The fully assembled digital structure undergoes a Finite Element Analysis (FEA) to assess its structural performance under simulated loads, allowing for optimisation before any physical fabrication begins.

### Fabrication

Step 7: Construction and fabrication processes commence using the evaluated designs from FEA. This phase incorporates MR-assisted assembly techniques to enhance precision and efficiency. The MR environment guides the assembly process, ensuring accurate placement and alignment of wooden components. Cornucopia Wooden House - The culmination of the process is constructing a wooden house, exemplifying the practical application of the SBDC framework. This step demonstrates the feasibility of upcycling irregular wood into viable, sustainable building structures.

### Material reclamation system (MRS)

#### Digital material database

**Fig. 5:**
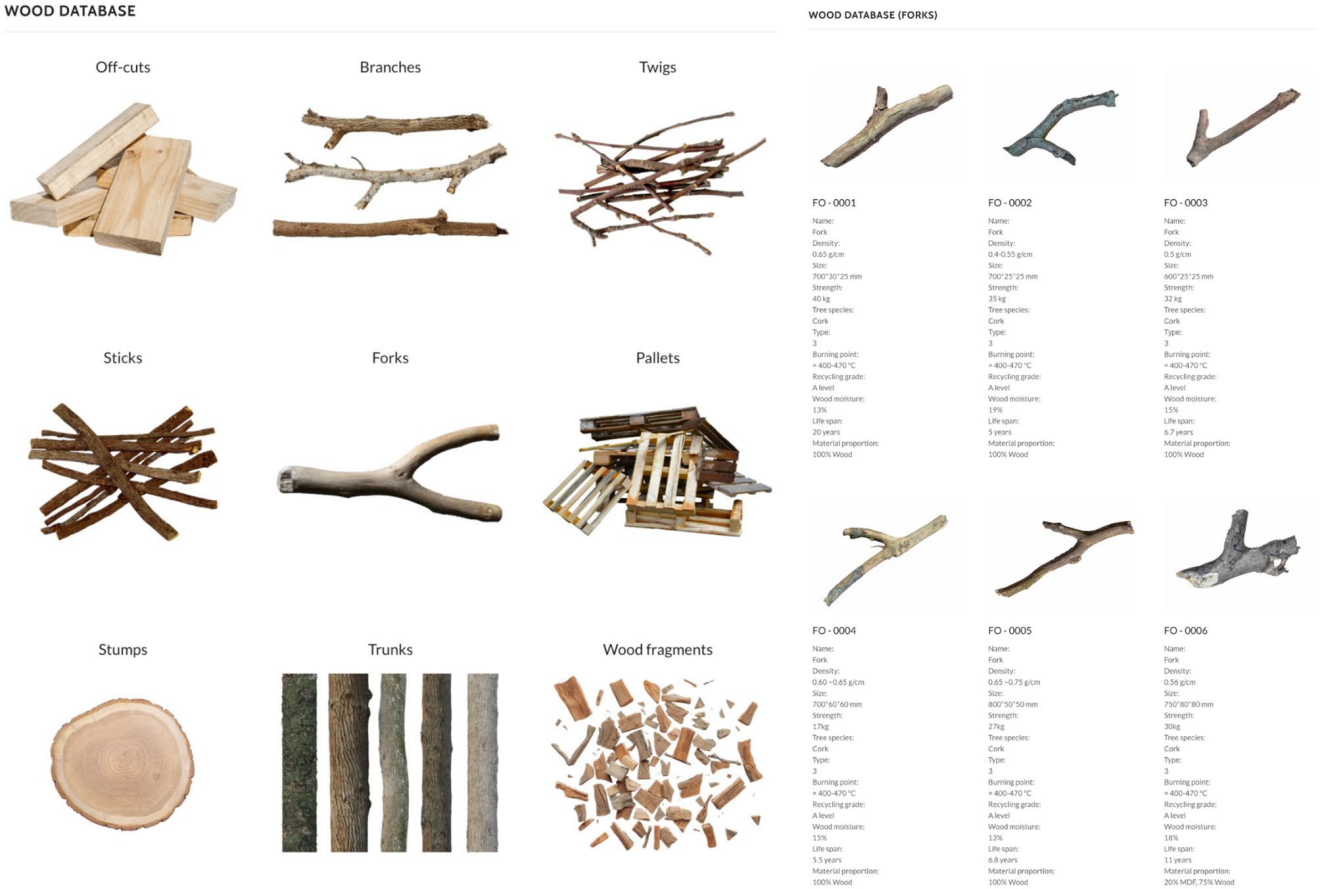
MRS material database (detailed online database: https://crowleyluo.com/wood-database*).*

The Material Reclamation System (MRS) conducts systematic collection and data acquisition of various wood types, including logs, trunks, forks, branches, and off-cut wood. Using flexible scanning technologies and storing detailed data (Fig. 4). The results are organized into four main sections: material collection, data acquisition, and data classification, specifically:

A. Material Collection: The collection phase involved sourcing wood from multiple origins, ensuring a diverse and comprehensive inventory. The primary sources included: (a) Furniture and Domestic/Industrial Architectural Elements: These provided used wood that, although previously employed in construction or manufacturing, retained structural integrity suitable for reclamation; (b) Twigs and Branches: Sourced from landscaping and pruning activities, these materials represent waste wood typically discarded but repurposed in our study; (c) Demolition and Pallet Wood: Derived from dismantled structures and shipping pallets, these materials often come in irregular shapes and sizes and are categorized as waste wood; (d) Sawmill and Factory Off-cuts: These are remnants from industrial processing, presenting a variety of wood types and forms that would otherwise be considered idled wood.
B. Data Acquisition: The process employed advanced 3D scanning technologies to capture the wood samples’ detailed geometric and physical characteristics. This step was crucial for creating accurate digital models, which formed the basis for subsequent design and fabrication processes. Divided into Large-scale Scanning, mesoscale scanning, and small-scale scanning, capturing fine details and textures essential for accurate digital representation. Also, the captured data included dimensions, shapes, surface textures, and material parameters such as density and moisture content. Measuring and recording the moisture content accurately, providing essential data for material classification.
C. Data Classification: Once acquired, the data for each individual wood element is classified based on its unique geometric and physical characteristics. This classification was essential for organizing the data into a structured database, facilitating easy retrieval and analysis. Each classified wood element is then represented as a collection of voxels within a larger 3D input matrix. This collection encodes the specific material type and its properties. These geometric features and material parameters included measurements of dimensions, shapes, and surface textures. These encompass densities, moisture content, and structural integrity. Here, structural integrity refers to a quantitative assessment of the wood’s condition, focusing on physical defects that could impact its load-bearing capacity. The measuring process involves identifying and quantifying defects such as knots, cracks, rot, and holes using the 3D scan data and visual inspection. The size, quantity, and location of these defects are recorded. This physical assessment is translated into a strength reduction factor (k_def_). This factor is a dimensionless value, typically ranging from 0 (no structural capacity) to 1 (pristine, no defects). For example, a piece of wood with minor knots might be assigned k_def_=0.8, indicating it retains 80% of its nominal strength. Accurately measuring these parameters ensured that the reclaimed element meets the required safety and performance standards for its intended use. Accurately measuring these parameters ensured that the materials met the necessary construction-use standards.

### Structure generation and assessment system (SGAS)

*How can we utilise the irregular wood and complex geometric features from a new perspective on AEC?*

A voxel-based design method could be a solution (Luo, 2024). Each voxel represents a unit of material with specific properties. The core of the SGAS is a modified Wave Function Collapse (WFC) algorithm, a procedural generation technique adapted for graphics and design. It works by starting with an empty grid where every location holds all possible states. The algorithm then iteratively “collapses” the state of one location to a single, definite choice. This choice then constrains the possibilities for adjacent locations based on a set of predefined rules, propagating throughout the grid until a complete, coherent assembly is generated from the database of unique reclaimed wood pieces. In this process, each piece of wood is first converted into a discrete voxel model, where each voxel contains data on the material’s properties such as species, density, and its structural integrity factor. The designer then defines a set of rules for the WFC algorithm, including adjacent constraints for how pieces connect, structural constraints for load paths, and geometric constraints governing orientation, which are optimized using a voxel rotation mechanism. The WFC algorithm then begins with an empty 3D grid and iteratively selects a position, collapsing its state from all possibilities to a single piece that satisfies the local constraints, propagating this choice to generate a coherent assembly. The generated design is then exported as a digital model for performance analysis using tools like Rhinoceros^®^¹ and Grasshopper^®^¹, where a Finite Element Analysis (FEA) is conducted to assess its response to various loads. The results of the FEA provide direct feedback to the designer, who can refine the WFC constraints and regenerate the structure in an iterative loop until the desired performance is achieved.

¹ Rhinoceros^®^ and Grasshopper^®^ are registered trademarks of Robert McNeel & Associates.

### Reclaimed irregular wood-based sustainable furniture design

Understanding the geometry and topology of irregular woods—often discarded due to their non-standard shapes—unlocks new opportunities for architectural and construction innovations. Notably, a non-degradative reclamation approach, which preserves original wood geometry with minimal processing, is rarely addressed in the literature. Furthermore, the inherent textural qualities and unique geometric topology of reclaimed timber are frequently overlooked during reuse. Research by Piccardo and Hughes^42^ highlights that many salvaged wood elements retain significant structural and aesthetic value but are underutilized because conventional systems fail to account for their irregularity. By integrating these rarely–considered material attributes into our workflow, we aim to elevate irregular wood from waste to resource—valuing its natural form, texture, and topology throughout design and construction.

To satisfy the non-degraded recovery of irregular wood, its geometric language and material are directly used to generate it within a given paradigm.

**Fig. 6:**
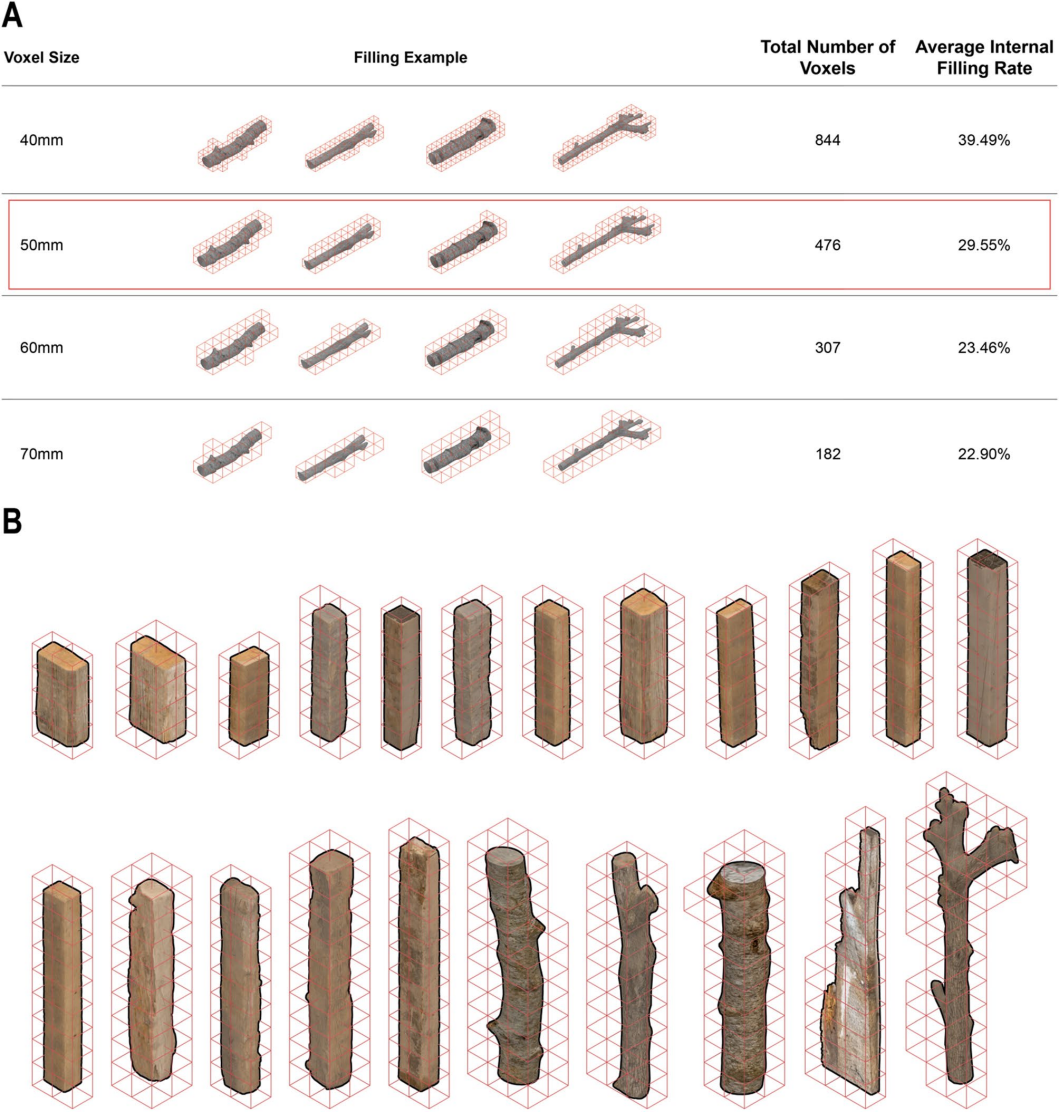
Voxel definition.

Voxel Size Definition (Fig. 6A): Irregular wood was voxelized, with the four most irregular examples selected for analysis. These were placed within voxels of 40 mm, 50 mm, 60 mm, and 70 mm side lengths. A comparative assessment of filling patterns, total voxel counts, and internal fill ratios revealed that the 50 mm voxel size yielded optimal results: an average total of 476 voxels and an internal fill ratio of 29.55%, both superior to other sizes. Consequently, 50 mm was selected as the experimental unit size. Input Matrix (Fig. 6B): All irregular wood pieces were voxelized at 50 × 50 × 50 mm to visualize proportional differences and voxel count variations.

Valid Joint Surface (Fig. 3A1): We define a valid joint surface as any voxel face where wood occupies > 50% of the surface area. Preferred Joint Surface (Fig. 3C2): A preferred joint surface is a valid joint surface that either occurs naturally as a flat plane or has undergone minimal processing (one machining operation). Closest Point Connection Rules (Fig. 3D1-2): For potential preferred joint surfaces, the midpoint of each outer frame face post-voxelization is identified. The distance between these midpoints and the internally filled wood voxels is computed. The voxel face with minimal distance is selected to define complementary connections between adjacent wood blocks.

**Fig. 7:**
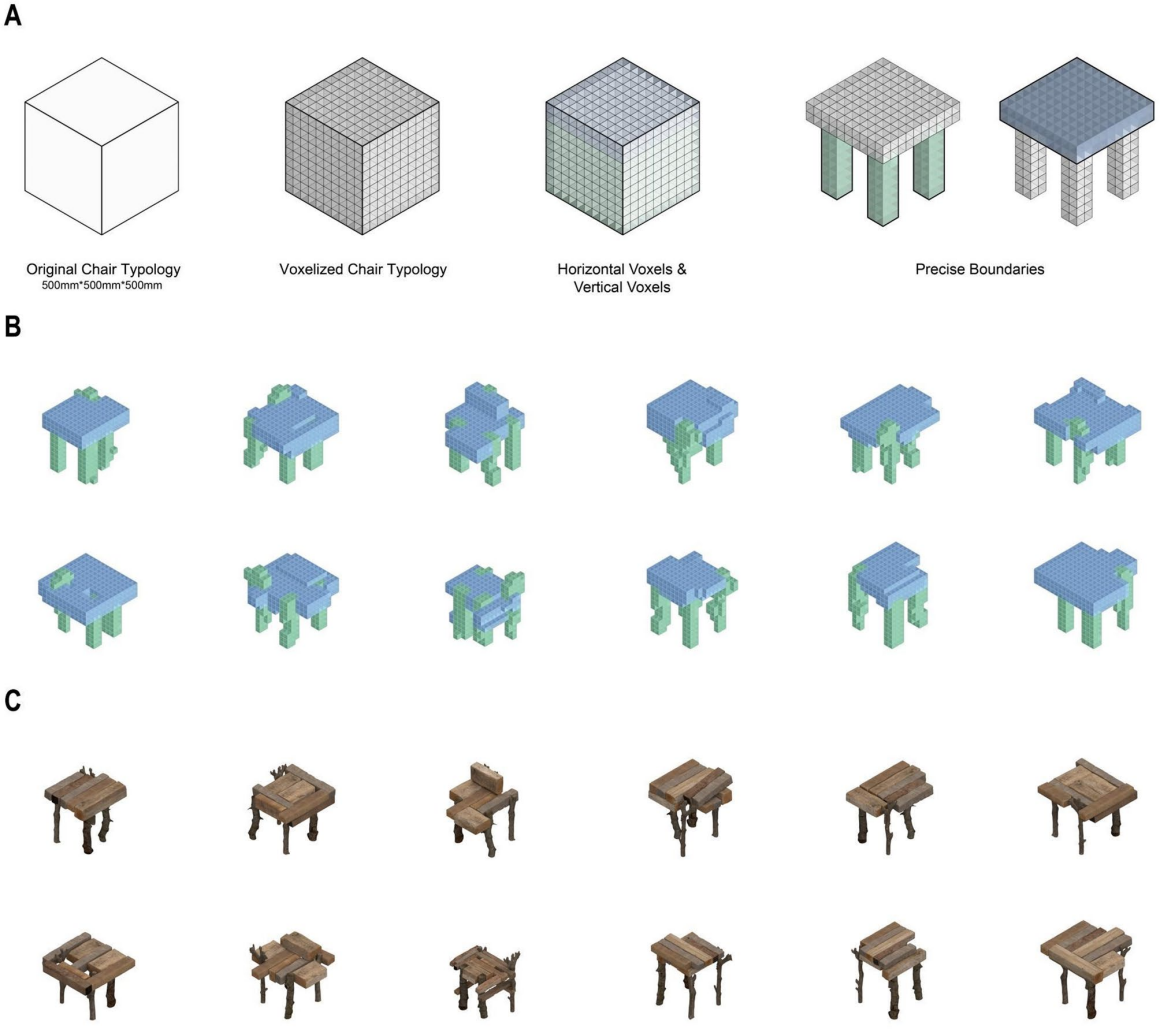
Generative design workflow for chair typology. (**A**) The initial setup, showing the voxelized design space with boundaries defined for the seat and leg substructures. (B-C) Two groups of distinct chair prototypes generated by the Wave Function Collapse (WFC) algorithm, which assembles components from the database to fit the constraints established in (A).

The structural generation process for the chair typology (Fig. 7) commences within a 500 × 500 × 500 mm voxelized bounding box. To achieve precise formal control, this volume is logically decoupled into functional substructures, such as the upper horizontal seat and the lower vertical legs. The Wave Function Collapse (WFC) algorithm is then applied to populate this constrained space, procedurally selecting and arranging irregular wood voxels from the database to create a complete digital chair model, adhering to distinct rules for each substructure to ensure a functional assembly.

**Fig. 8:**
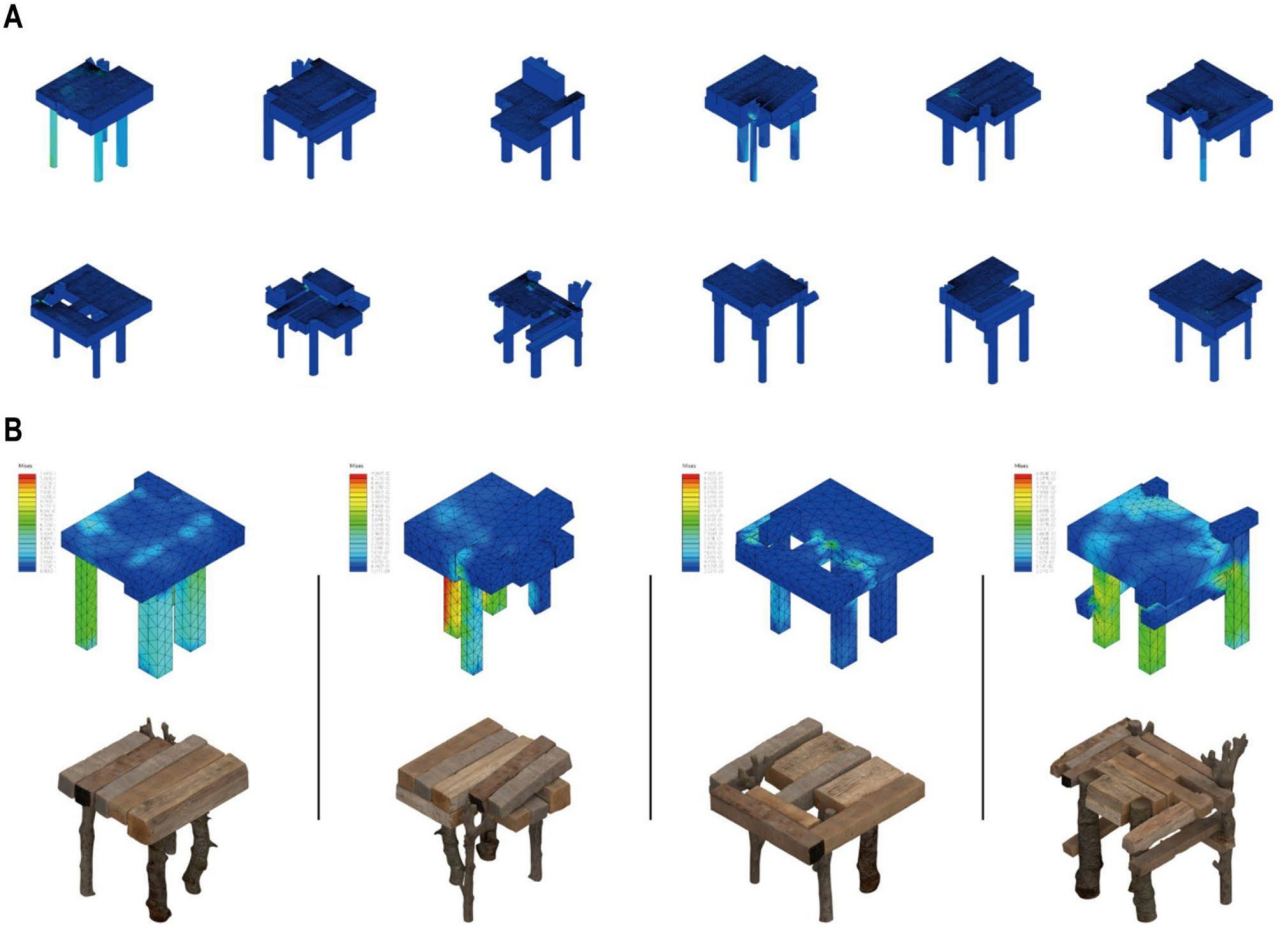
Structural Validation and Design Iteration via FEA. (**A**) The FEA stress analysis of an initial prototype reveals structural situation. (**B**) Revised prototypes, generated with the new rules, is shown to successfully meet basic structural criteria.

The digital chair generated by the WFC serves as the direct input for a structural validation stage within an iterative design loop (Fig. 8). This stage uses Finite Element Analysis (FEA), acts as a validation tool for the spatial optimization performed by the SGAS, to verify the design against specific engineering criteria, ensuring its structural viability before fabrication. The analysis is configured with specific boundary and load conditions: the base of the chair legs is fully fixed to simulate floor contact, and a standard static load of 1.08 kN^25^ is applied to the seating surface. The FEA is directly linked to the classified capacity of each unique wood element. This directly addresses the question of whether an element can support the calculated mechanical stress by evaluating it against the specific estimated capacity of each piece of reclaimed wood.

A design is considered successful only if it meets all predefined criteria under the simulated load. These include a stress criterion, where the maximum calculated stress within any element must not exceed its unique allowable stress value; a deflection criterion, requiring the maximum vertical deflection of the seat to remain below a serviceability limit of 5 mm; and a stability criterion, where the leg structures must exhibit a buckling load factor greater than 2.0. If a design fails, the designer uses this specific feedback to refine the WFC constraints, for instance, by adding a rule to enforce the selection of elements with a higher k_def_ in that region. The WFC algorithm is then re-run to generate an improved design, and this iterative loop continues until a design meets all success criteria (Fig. 8B), validating it for fabrication.

### Design-to-fabrication process for creating furniture from reclaimed irregular wood

AR displays prior to fabrication are used to show designs at 1:1 in the real environment and their adaptability to surrounding objects. This allows for an initial assessment of the design’s presence and its relationship to the human body, use in space and experience.

Following this visualisation step, the fabrication phase employed the MR-assisted assembly method detailed in Sect. 3 to construct the furniture prototypes. This approach was chosen to test the framework’s ability to provide accessible and intuitive guidance for non-expert users. Using a Microsoft HoloLens running Fologram, digital models of the selected chair design were overlaid as 1:1 scale holograms in the physical workspace. This provided intuitive, step-by-step guidance, transforming the assembly from a task of interpreting abstract 2D drawings to a direct, tangible process of matching physical components to their holographic counterparts. This makes it particularly accessible to people who don’t have the training to understand construction drawings.

**Fig. 9:**
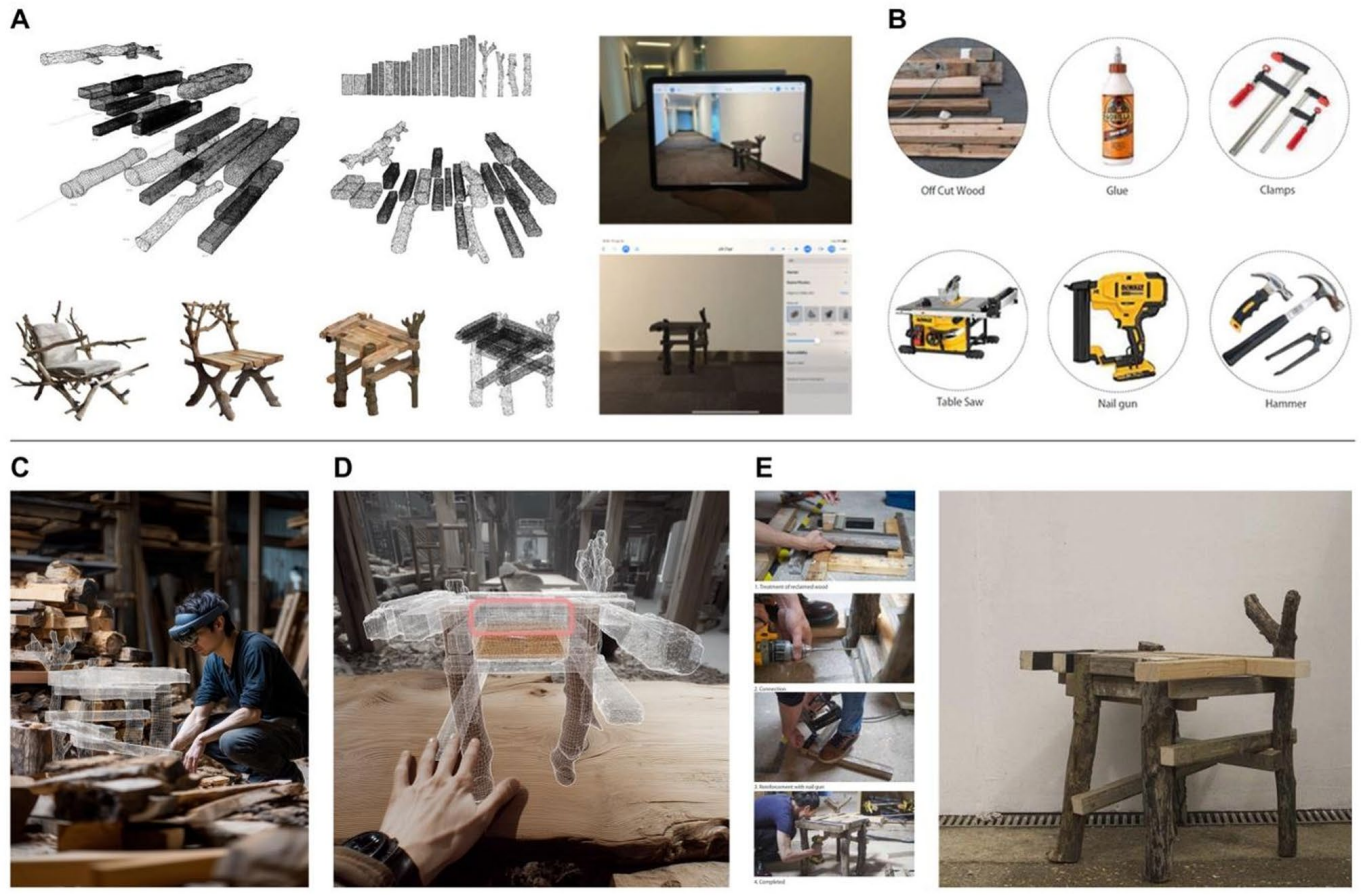
MR-Assisted Furniture Assembly. (**A**) Material and design proposal selection. (**B**) Tools for assembly. (**C**) A non-expert user wearing a HoloLens headset views the holographic model of a chair component overlaid in the physical workspace. (**D**) A screenshot from the user’s perspective, showing the digital overlay guiding the precise placement of an irregular wooden leg. (**E**) The physical assembly process and the completed chair.

**Fig. 10:**
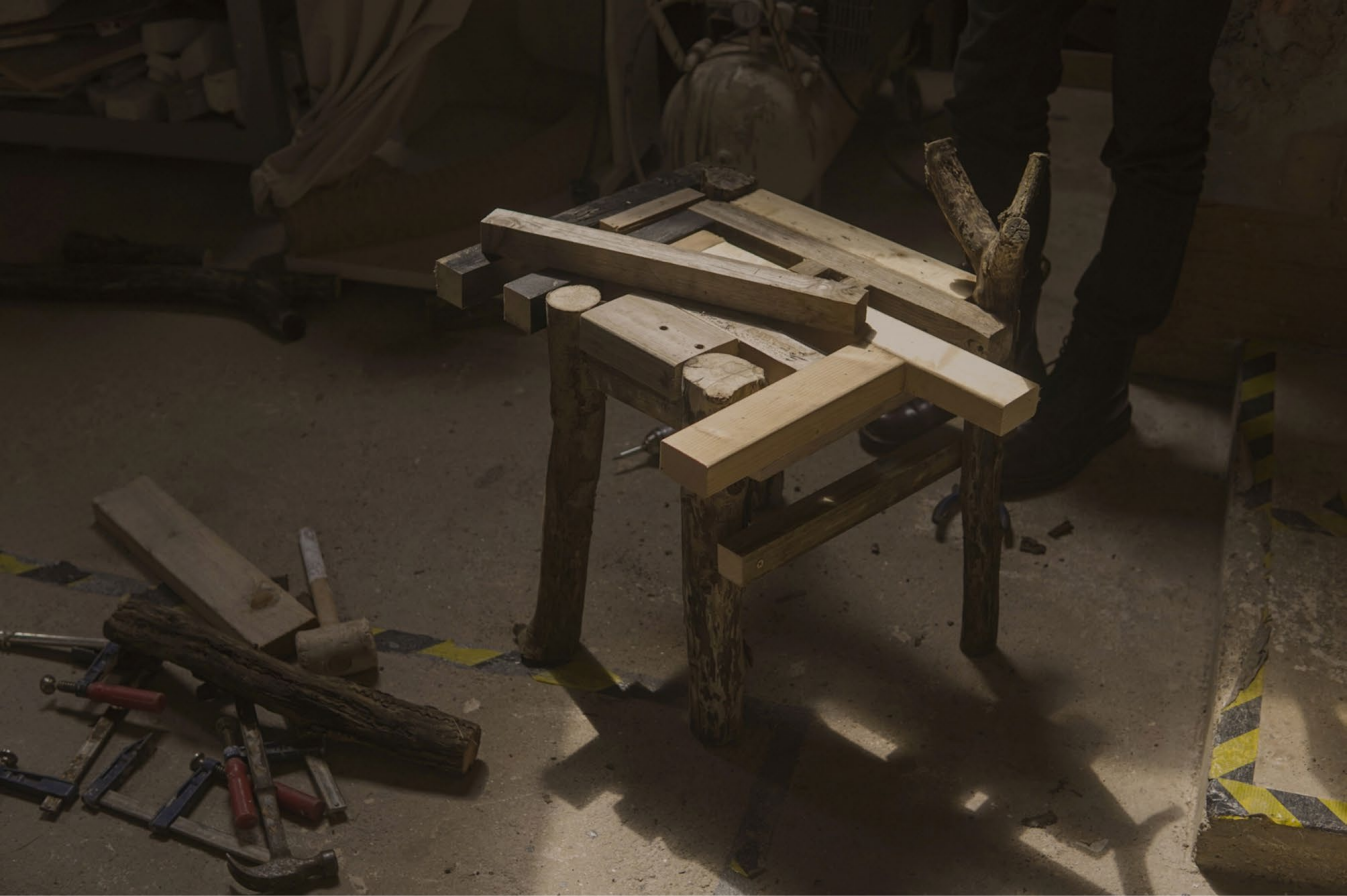
Completed chair assembly.

As illustrated in Fig. 9, the user could see the precise target location and orientation for each irregular log and trunk, allowing them to select the correct piece from the physical inventory and align it with its hologram (Fig. 9C). This process of direct visual matching significantly reduced the complexity and potential for error inherent in assembling non-standard components. The system guided the user through the assembly sequence, displaying the hologram for the next component only after the previous one was correctly positioned, as shown from the user’s first-person perspective in Fig. 9D. This achievement demonstrates the primary focus of our MR integration (Fig. 10): facilitating the assembly of complex, non-standard components by lowering the barrier to entry for users without specialised carpentry skills.

## A heat-moldable wood joinery method using repurposed plastic bottles

Recycled plastic bottles can serve as an innovative, heat-moldable joinery material for wood, transforming waste into a functional and sustainable resource. When heated, a standard PET beverage bottle shrinks tightly around wood components, binding them together without the need for adhesives or mechanical fasteners. In this technique, rings cut from discarded bottles function like large heat-shrink tubes, effectively locking wood joints in place. The plastic conforms to and grips the irregular surfaces of the wood, meaning that the joint’s strength benefits from natural surface roughness and grooves. As a result, this method enables the creation of reliable joints even for users lacking specialized woodworking skills or equipment.

**Fig. 11:**
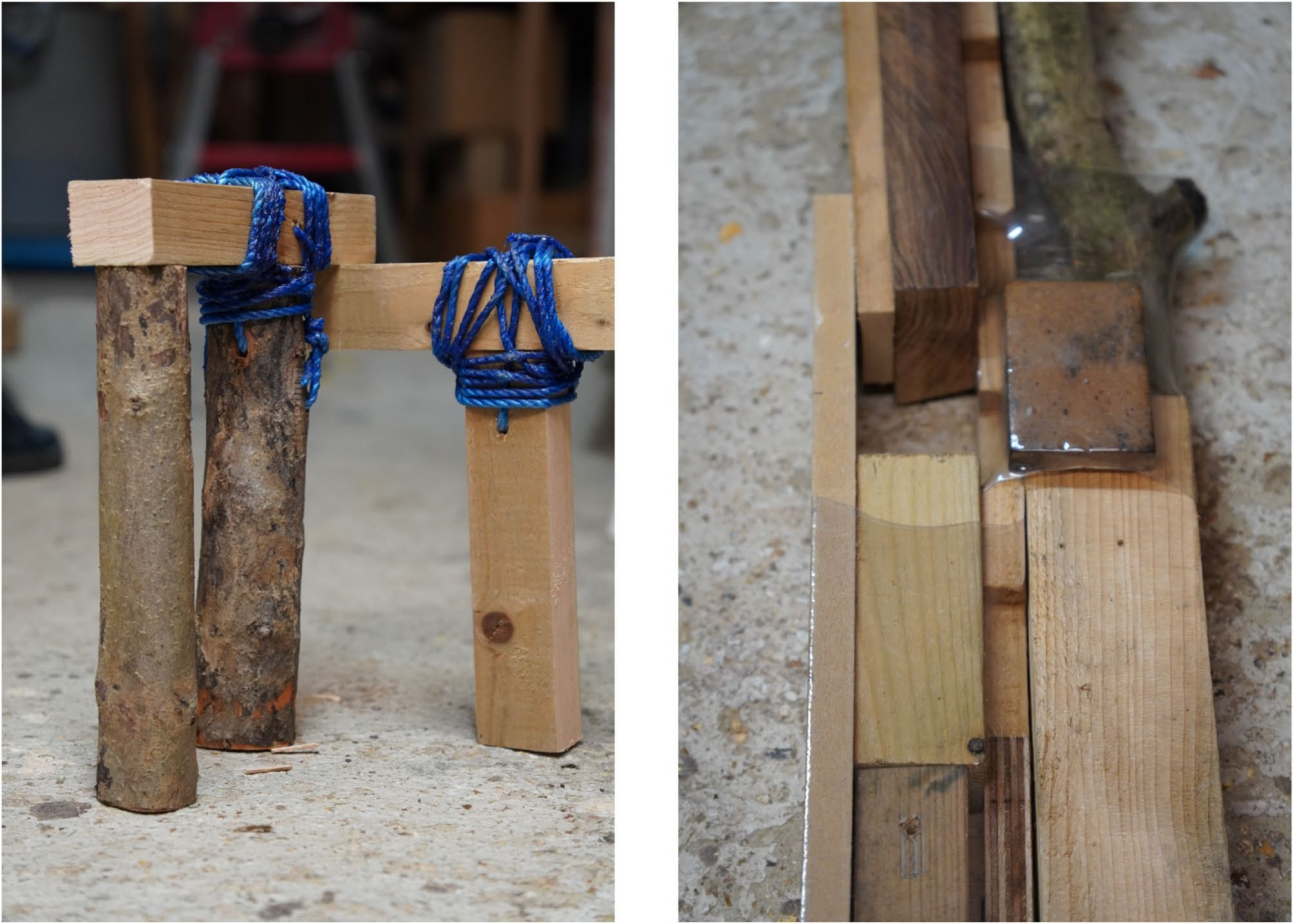
Comparison works with plastic bottles and rope joints.

This technique applies particularly to reclaimed wood construction, where builders often contend with miscellaneous pieces salvaged from deconstructed buildings. These components are typically irregular in size, partially damaged, or otherwise unsuitable for conventional joinery. Heat-shrink plastic joinery offers a low-impact solution for assembling reclaimed beams or planks into new configurations without requiring extensive re-milling. For instance, a non-structural partition wall or a pavilion roof could be constructed from short salvaged boards by overlapping them and securing them at intervals using shrink-wrap rings. This approach is analogous to lashing poles together with rope—except here, the “rope” is transparent plastic (Fig. 11). In scenarios where nails might split aged or brittle wood, shrink-wrapping provides a gentler alternative, distributing pressure evenly around the joint. Furthermore, it preserves the integrity and aesthetic character of historic or uniquely textured timber, eliminating the need for intrusive cutting to form traditional joints. Instead, components are externally bound, maintaining their original form.

### Fabrication process: heating, wrapping, and solidification techniques

**Fig. 12:**
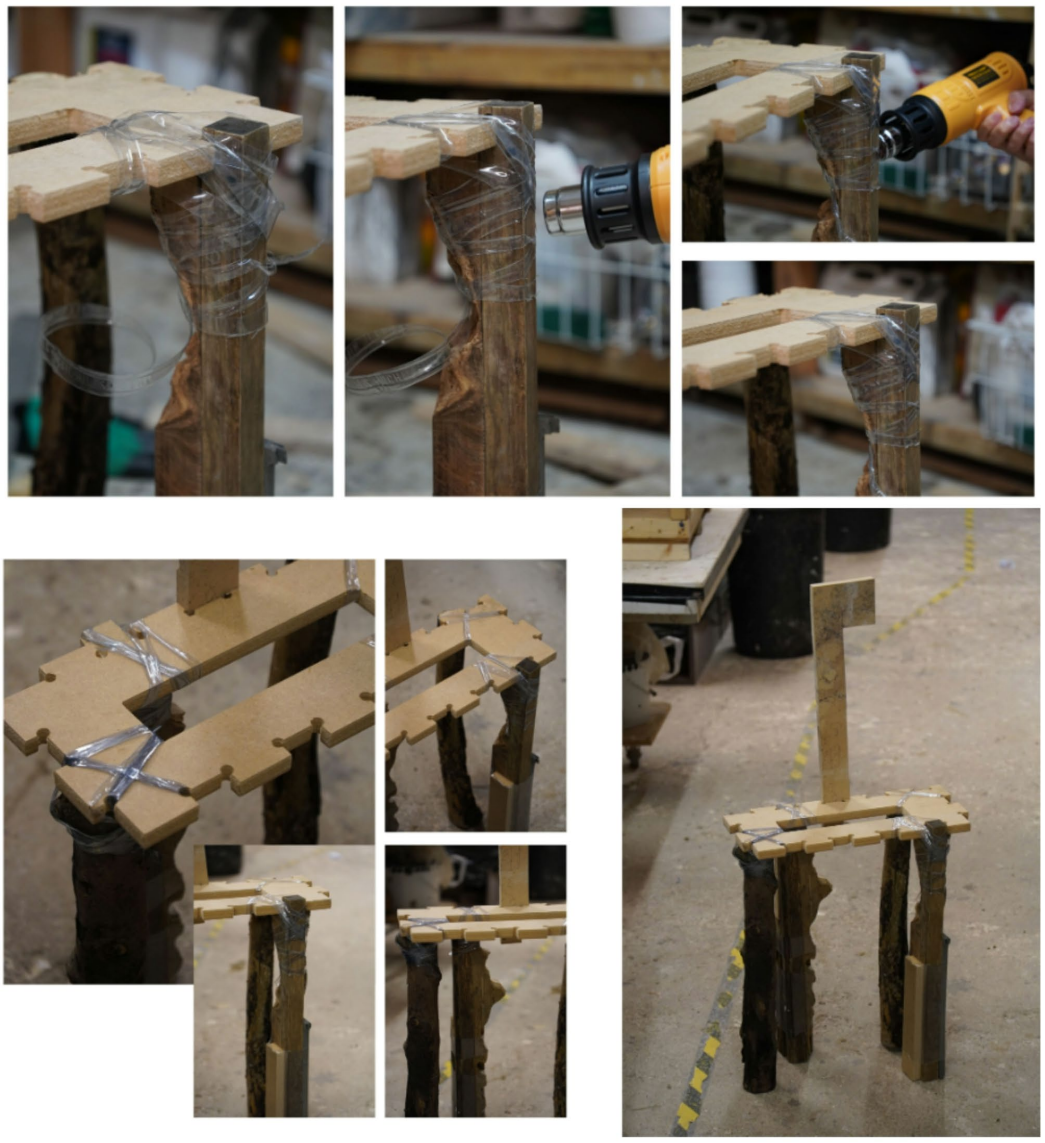
Plastic heat-moldable joinery design.

Using recycled plastic bottles for wood joinery involves three main stages: material preparation, heat activation, and cooling solidification (Fig. 12). These steps are outlined as follows:

Preparing the Wood: The wooden members to be joined (often reclaimed offcuts) are cut or arranged to fit together at the joint. It helps to carve small notches or grooves at the joining areas, since the strength of the joint depends on irregularities or grooves in the wood that the plastic can grip​. Flat mating surfaces are ideal; very complex angles or smooth round surfaces may not hold as well without some notching for grip​.

Preparing the Plastic Sleeve: A suitable recycled PET bottle is selected based on diameter – it should be just large enough to fit around the joint area before shrinking (the correct size bottle needs to be used for a good fit)​. The top and bottom of the bottle are cut off to produce a cylindrical sleeve or ring of plastic. This ring is essentially a piece of shrink-wrap, ready to connect the wood pieces.

Positioning and Wrapping: The plastic sleeve is slipped around the joint, encircling the two (or more) wood pieces that are being joined. The wood pieces are held in the desired alignment (either by hand, jig, or clamps) while the plastic is positioned. In some cases, multiple rings or a taller section of the bottle can be used to cover more of the joint for added strength.

Heating (Activation): A heat source is applied to the plastic evenly around the joint. Commonly, a heat gun set around ~ 300 °C is used to provide the necessary heat​. The hot air causes the PET material to soften and shrink dramatically, tightly conforming to the shapes of the wood underneath. As one designer explains, “taking a plastic bottle, cutting it, and then putting it around two pieces of wood… then I heat it so it shrinks and creates a joint^43^.”​ The plastic first contracts in diameter to clamp the wood, and shrinks longitudinally, pulling the wood pieces together like a tight band. Notably, even simple tools can accomplish this step – in a pinch, a hair dryer or other improvised heating element can suffice if carefully used on thinner plastic​ (though a proper heat gun yields more uniform results).

Solidification and Cooling: The heat source is removed once the bottle material has fully shrunk and the joint is snugly wrapped. The plastic quickly cools and re-solidifies in its new shape. PET transitions from a rubbery state back to a stiff solid as it cools, effectively “freezing” the wood joint in place. The result is a rigid plastic collar around the wood connection. After cooling, the joint is ready for use—unlike traditional adhesives, no curing time is needed beyond cooling.

Finishing (Optional): Once the joint is formed, any excess plastic or sharp edges can be trimmed or sanded. The aesthetic can be chosen by selecting different bottles—clear plastic can make the joint less visible, while colored bottles can make the joint a visual feature of the design​. The finished connection is a fusion of wood and recycled plastic, typically with a distinctive textured band where the bottle has gripped the wood.

### Architectural scale proposal - A pavilion design

In the architectural scale design and fabrication proposal, this project demonstrates an integrated workflow for circular architectural design using irregular, reclaimed wood components, guided by a cyber-physical design and decision-making process. The design begins with 3D scanning (supplementary video 3) and mesh reconstruction of diverse wood elements—including forks, off-cuts, and trunks—resulting in high-resolution digital models that capture geometric complexity. These scanned assets form the basis for a generative design process, aggregating components into a cohesive pavilion structure. The design prioritises circularity by leveraging the irregularity of reused materials as a generative constraint rather than a limitation.

**Fig. 13:**
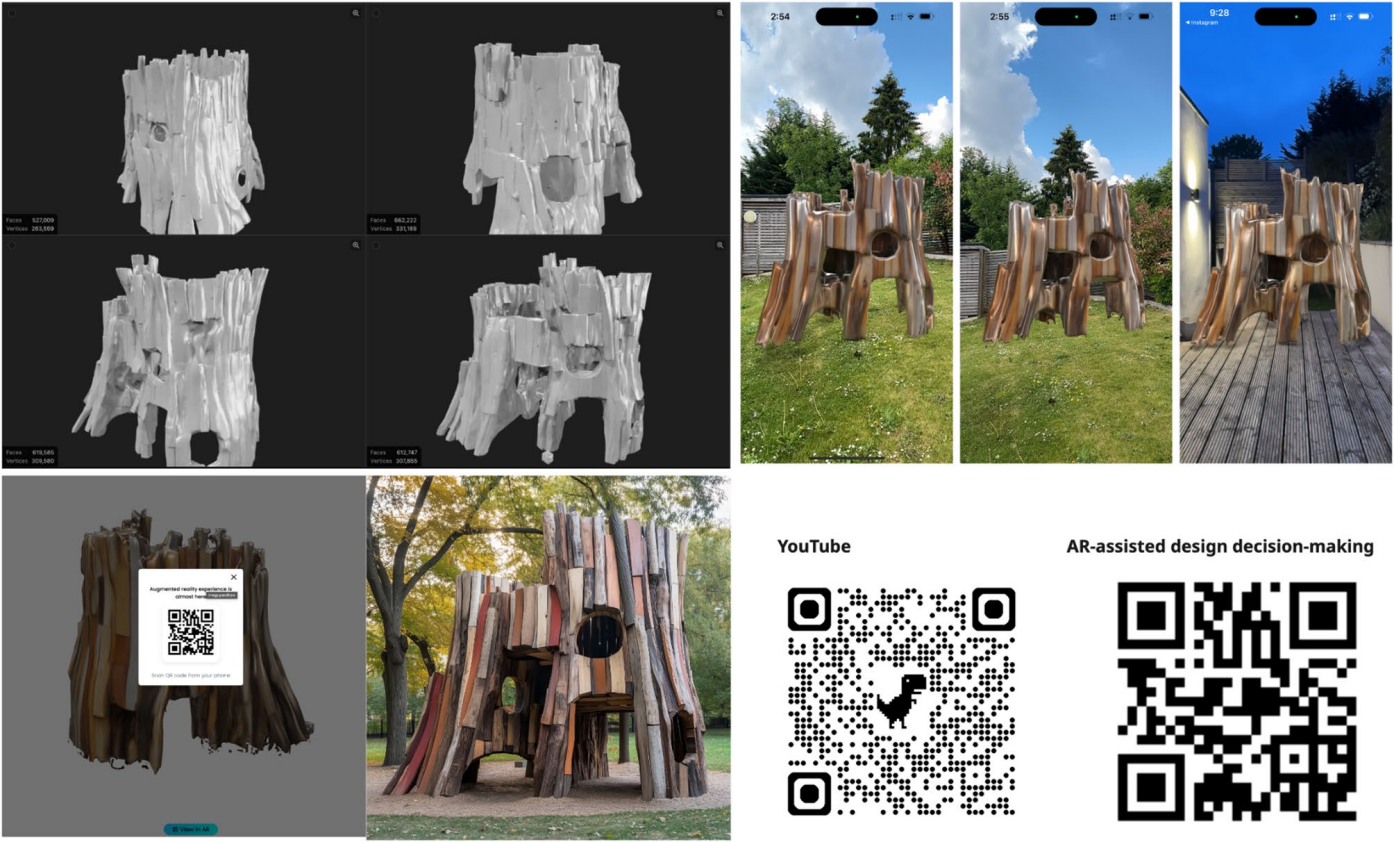
AR-assisted visualisation for design decision - Architectural Design Proposal (https://youtube.com/shorts/r5bm4xejy1c*)*.

Following digital modelling, the structure is deployed into an AR environment, enabling users to visualise the pavilion at full scale within various real-world contexts—daylight, evening, and urban settings—on mobile devices. This immersive AR experience facilitates participatory feedback and enhances design decision-making by allowing users to interact with the structure spatially and contextually. The inclusion of a scannable QR code enables broad accessibility, encouraging engagement from both professionals and the public. Furthermore, the project yielded a specific finding regarding the role of Augmented Reality (AR) in the design loop. After deploying the initial design into an AR environment for full-scale visualisation (Fig. 13), it became evident that an entrance, which appeared suitable in digital models, was spatially constricting and obstructed key sightlines when experienced in a real-world context. This insight, gained through the immersive AR experience, prompted a direct revision of the generative constraints to widen the entryway.

The final pavilion prototype reflects the accumulated digital-to-physical process, demonstrating the feasibility of assembling non-standard wood elements into architecturally meaningful forms. This AR-driven feedback loop demonstrates how non-experts, when empowered with intuitive, full-scale visualization, can provide valuable spatial and functional design input. It proves the efficacy of AR as a tool to bridge the gap between abstract digital models and human-centric experience, directly influencing the final architectural form and validating its real-world viability before fabrication. Furthermore, the integration of video documentation and public dissemination through platforms like YouTube extends the project’s educational and communicative reach.

## Discussion

Future research should address the limitations of current upcycling practices by expanding the scope of materials studied and developing comprehensive sustainability metrics through lifecycle assessments. Investigating a broader range of wood types and other natural materials can uncover new opportunities for material reuse and provide a more holistic understanding of sustainable construction. Developing robust sustainability metrics, including carbon footprint, energy consumption, and resource efficiency, will validate the environmental benefits of upcycling irregular wood. Integrating the upcycling framework with broader circular economy models can enhance its applicability, fostering innovation in material use and design. This integration emphasises minimising waste and maximising resource use through recycling and repurposing, aligning with circular economy principles.

**Fig. 14:**
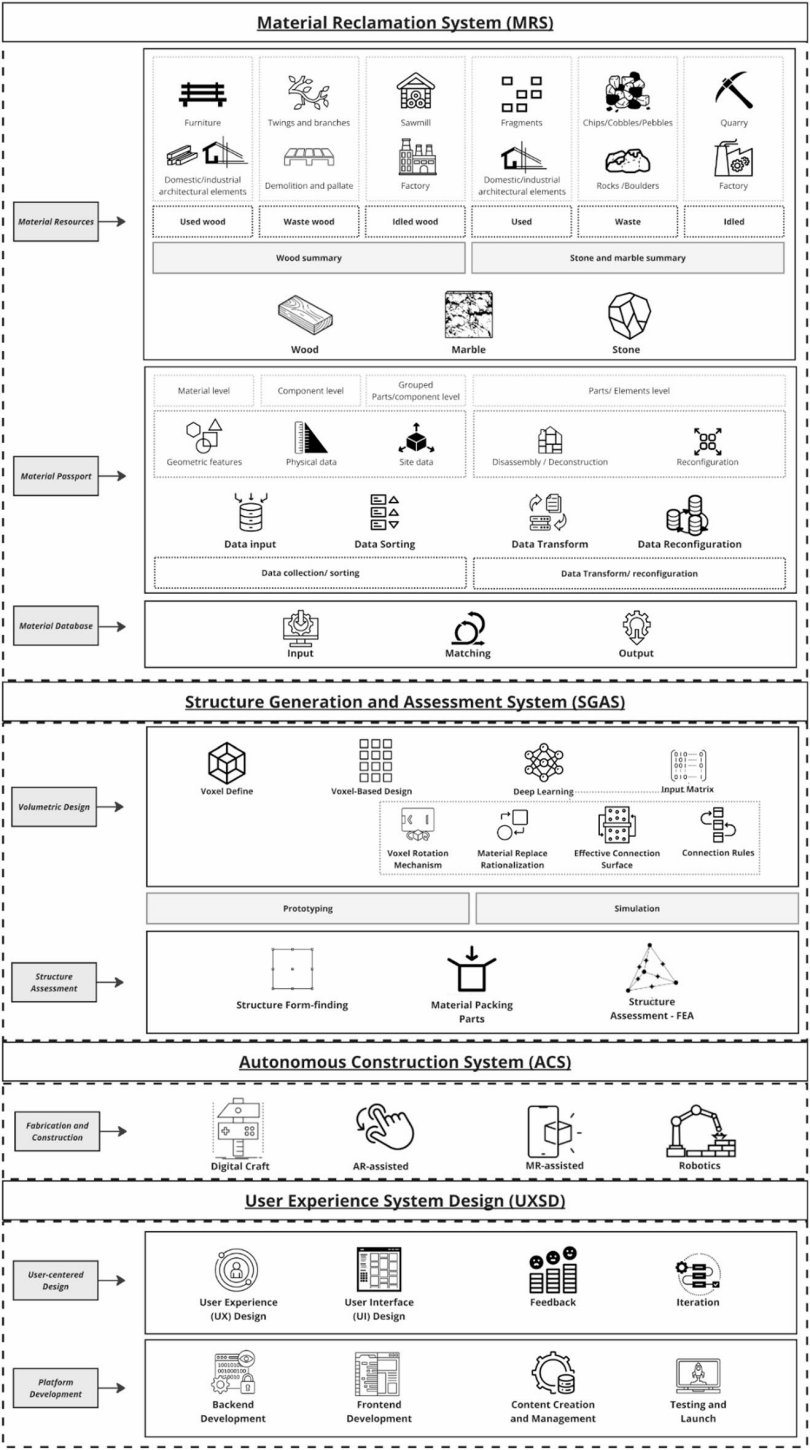
Integrating UXSD modules in SBDC.

 Methodological improvements are essential for advancing upcycling practices (Fig. 14). Enhancing data collection techniques through advanced 3D scanning technologies will ensure accurate capturing of irregular wood’s geometric and physical properties. Refining voxel-based methods can improve the accuracy of material placement and alignment, maintaining structural integrity and aesthetic appeal. Furthermore, the material classification system employed in this study (Material Hierarchy for Structural Potential) is primarily based on visual assessment of degradation and grain continuity from 3D scans. It does not incorporate formal, quantitative timber grading standards that assess mechanical strength, internal defects (e.g., hidden knots, decay), or precise fibre orientation, which are critical for structural safety in real-world applications.13 Future research should address this by integrating advanced non-destructive testing (NDT) methods, such as acoustic testing, stress wave analysis, or X-ray tomography, to obtain more accurate and comprehensive data on the internal mechanical properties and defects of irregular timber elements. This would allow for a more robust material database and enable FEA to be linked directly to the actual load-carrying capacity of each unique timber piece, moving towards certified structural applications. Optimising MR-assisted assembly for broader accessibility involves making these technologies more user-friendly and reducing the learning curve for users. Developing advanced machine learning models to handle wood variability will predict the best arrangement of wood pieces, improve efficiency and reducing waste. Conducting field trials in real-world settings will identify practical challenges and opportunities for improvement, demonstrating the economic and environmental benefits of upcycling.

One significant limitation in applying FEA to the structural assessment of reclaimed, irregular wood lies in the simplification of geometry during the simulation process. Due to the complex and non-standard shapes of reclaimed components, such as logs, forks, and off-cuts, precise geometric modelling is often not feasible within standard FEA tools. Instead, these elements are typically approximated using regularised or idealised geometries (e.g., cylindrical or voxel-based forms) to facilitate meshing and simulation. While this approach enables computational efficiency and allows for general stress distribution analysis, it inherently overlooks local irregularities, surface imperfections, and anisotropic grain directions that significantly influence the actual structural behaviour of wood. As a result, the accuracy of the simulation outcomes may be limited, particularly in predicting local failure zones, joint behaviour, and contact interactions between uniquely shaped components.

The success of plastics as structural connectors at the home scale does not mean that they are feasible at the building scale. Therefore, after the prototype development test, the next step will be to conduct further prototype testing on high-performance engineering plastics based on the tensile stress load and other requirements of the building scale, using engineering plastics such as polycarbonate (PC), nylon (PA), glass fiber reinforced plastic (GF-reinforced polymer) with good strength, toughness and impact resistance. And according to the purpose, plastics with flame retardant, UV protection, corrosion resistance and other properties can be selected. Potential use: Nylon + 30% glass fibre is often used in 3D printing structural joints, and its performance is close to that of aluminium. This method, while promising for certain applications, currently lacks the rigorous structural and durability testing required for building-scale applications. Future research should focus on conducting comprehensive prototype testing on high-performance engineering plastics, such as polycarbonate (PC), nylon (PA), or glass fibre-reinforced polymers, to assess their tensile stress load, shear strength, and other mechanical properties relevant for building-scale requirements. Additionally, critical aspects such as durability in various environmental conditions (e.g., moisture, UV exposure, temperature fluctuations) and fire retardancy must be thoroughly investigated. The potential for moisture entrapment within timber elements when using plastic connections, which could lead to rapid degradation due to microorganisms in humid climates, necessitates extensive long-term durability studies.

While the integration of MR in design and construction workflows promises increased accessibility and intuitive guidance for non-expert users, the assumption that MR inherently ensures precision for unskilled participants remains largely unverified. The claim is compelling—MR overlays can visually instruct users on positioning, sequencing, and orientation—but actual execution depends on multiple variables that are not always accounted for. These include device tracking accuracy, user interpretation of visual cues, real-world material tolerances, and environmental conditions such as lighting or spatial constraints. Without empirical evaluation, such as controlled experiments comparing MR-guided tasks by skilled versus unskilled users, the effectiveness of MR in delivering construction-level precision remains speculative. The current study successfully demonstrates MR’s value as a participatory and instructional tool by enabling a non-expert to complete a complex assembly; however, it does not provide the quantitative user studies needed to validate its reliability for achieving certified construction-level precision, which remains a key area for future research.

Collaboration with industry partners, educational programs, and community involvement is crucial for promoting the adoption of sustainable practices. Industry partnerships can facilitate knowledge exchange, innovation, and the development of standardised practices and guidelines. Educational programs, including workshops and certification courses, will equip construction professionals with the necessary skills and knowledge to implement upcycling methods. Involving stakeholders and the community through awareness campaigns and participatory design workshops will foster local participation and support^44^. Future research should also focus on policy and regulatory support, advocating for incentives and standards that promote sustainable construction. Comprehensive evaluation methods, including cost-benefit analyses and social impact assessments, will provide a holistic understanding of the advantages and challenges of upcycling. These efforts will ensure that innovative upcycling methods become practical, impactful, and widely adopted, contributing to a more sustainable and resilient built environment.

Specifically, we have added a quantitative comparison in the future between conventional CNC-based rectilinear material preparation and our AI-driven aggregation method using Wave Function Collapse (WFC). This includes metrics such as:

Material Utilisation Rate (%): We calculate the proportion of usable volume retained from raw irregular logs before and after the design aggregation process. In our case studies, the WFC-based method increased material utilisation by an average of 28–35%, compared to conventional milling approaches.

Offcut Reduction (kg or m³): We estimate the volume of residual wood left unused after layout optimisation. The WFC method minimises the need for standardised trimming, thereby decreasing offcut waste significantly.

Time-to-Fit Efficiency (hrs/unit): We report a reduction in the time required to select and adapt wood components through automated fitting, contributing to more efficient assembly and less discarded trial material.

## Conclusion

This study successfully introduces and demonstrates an AI-driven computational workflow for the upcycling of irregular timber, addressing the critical challenge of material underutilisation and waste in sustainable building design and construction. By integrating advanced digital technologies—including precise 3D scanning for material capture, the Wave Function Collapse (WFC) algorithm for automated spatial aggregation, and Finite Element Analysis (FEA) for iterative structural assessment—the framework provides a robust methodology for transforming complex, non-standard timber elements into valuable components for architectural applications. The application of WFC as a generative design tool effectively manages material variability, facilitating optimal spatial configurations and significantly enhancing material utilisation rates while reducing offcut waste.

Furthermore, the integration of Mixed Reality (MR) guidance for visualisation and assembly demonstrates a powerful approach to democratising fabrication processes, making complex assembly tasks more accessible to a wider range of users. The successful fabrication of functional furniture and pavilion-scale architectural prototypes underscores the practical applicability and scalability of this framework. While limitations regarding comprehensive material characterisation for certified structural performance and empirical validation of MR precision in this specific context are acknowledged, this research lays a strong foundation for future advancements. This work significantly contributes to Computer-Aided Architectural Design (CAAD) and construction techniques by providing a repeatable and scalable solution for circular material reuse, ultimately fostering a more sustainable and resource-efficient built environment.

## Supplementary Information

Below is the link to the electronic supplementary material.

Supplementary Material 1

## Data Availability

The datasets generated during and/or analysed during the current study are available from the corresponding author on reasonable request.
