# Supplementary material for: Upcycling of regular wood trunks and logs using wave function collapse (WFC), augmented reality (AR), and mixed reality (MR) technologies for circular design
Source: Sci Rep. 2025 Oct 21;15:36599. doi: 10.1038/s41598-025-20398-8 (PMC12541094; doi:10.1038/s41598-025-20398-8)
Supplement: Supplementary file 1 — Supplementary Material 1 [file 41598_2025_20398_MOESM1_ESM.pdf]

Supplementary Materials

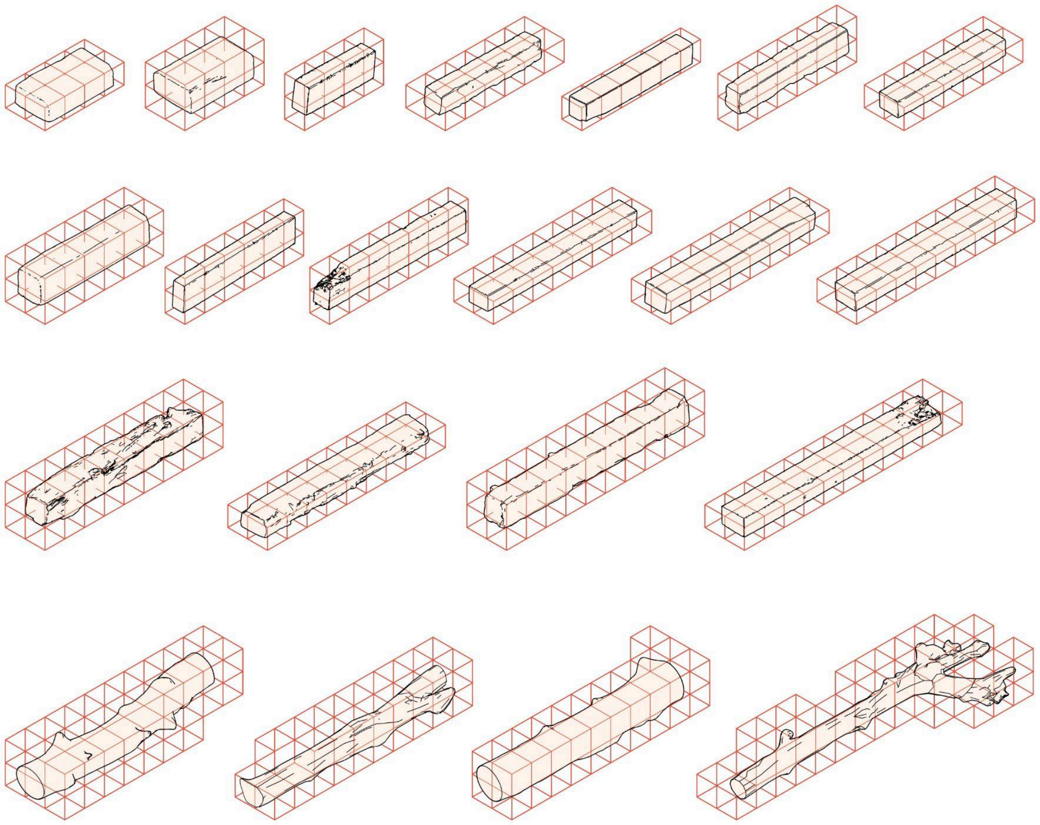

Supplementary Figure 1. Voxelized irregular wood pieces.

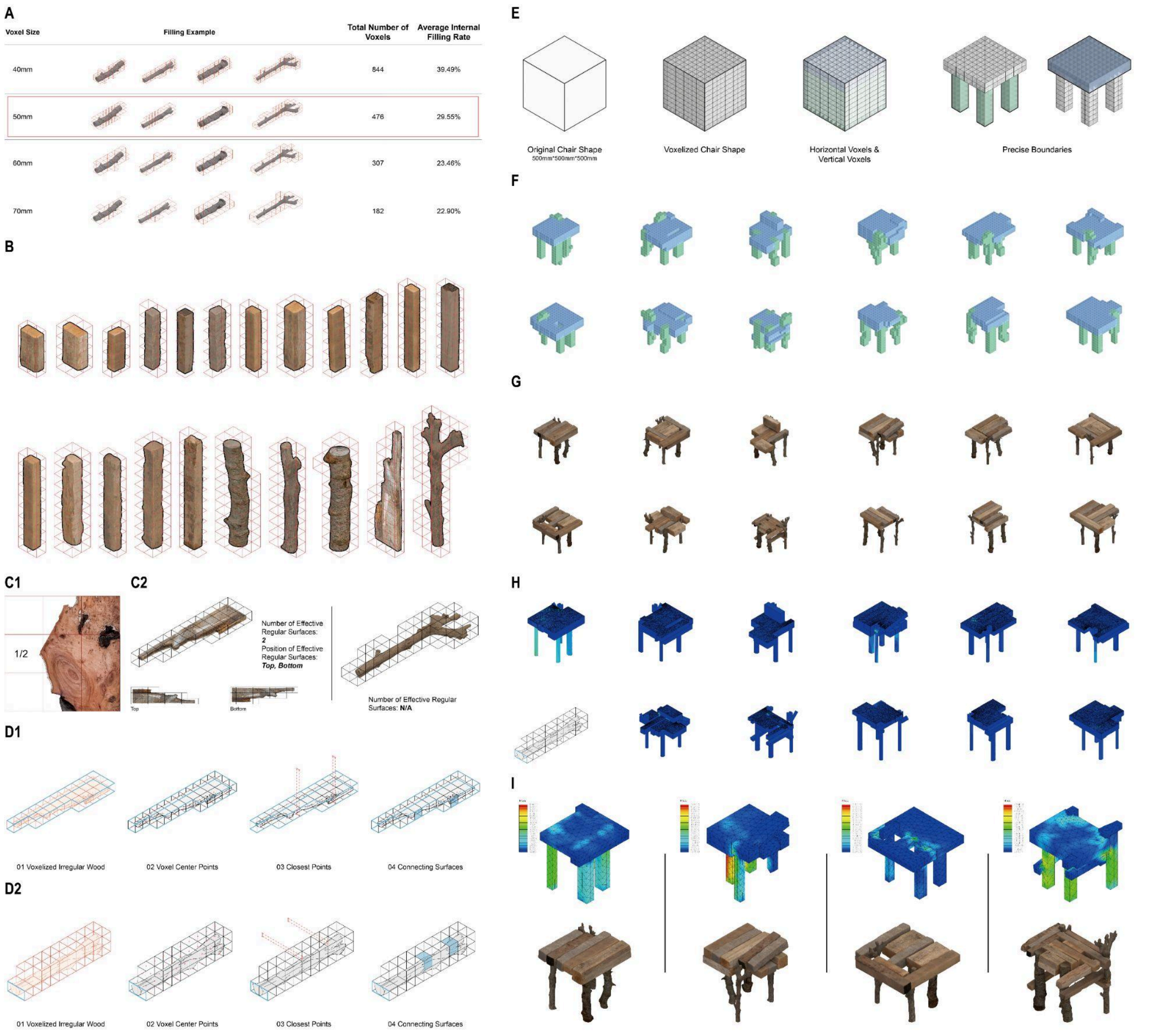

Supplementary Figure 2. Reclaimed irregular wood-based sustainable furniture design.

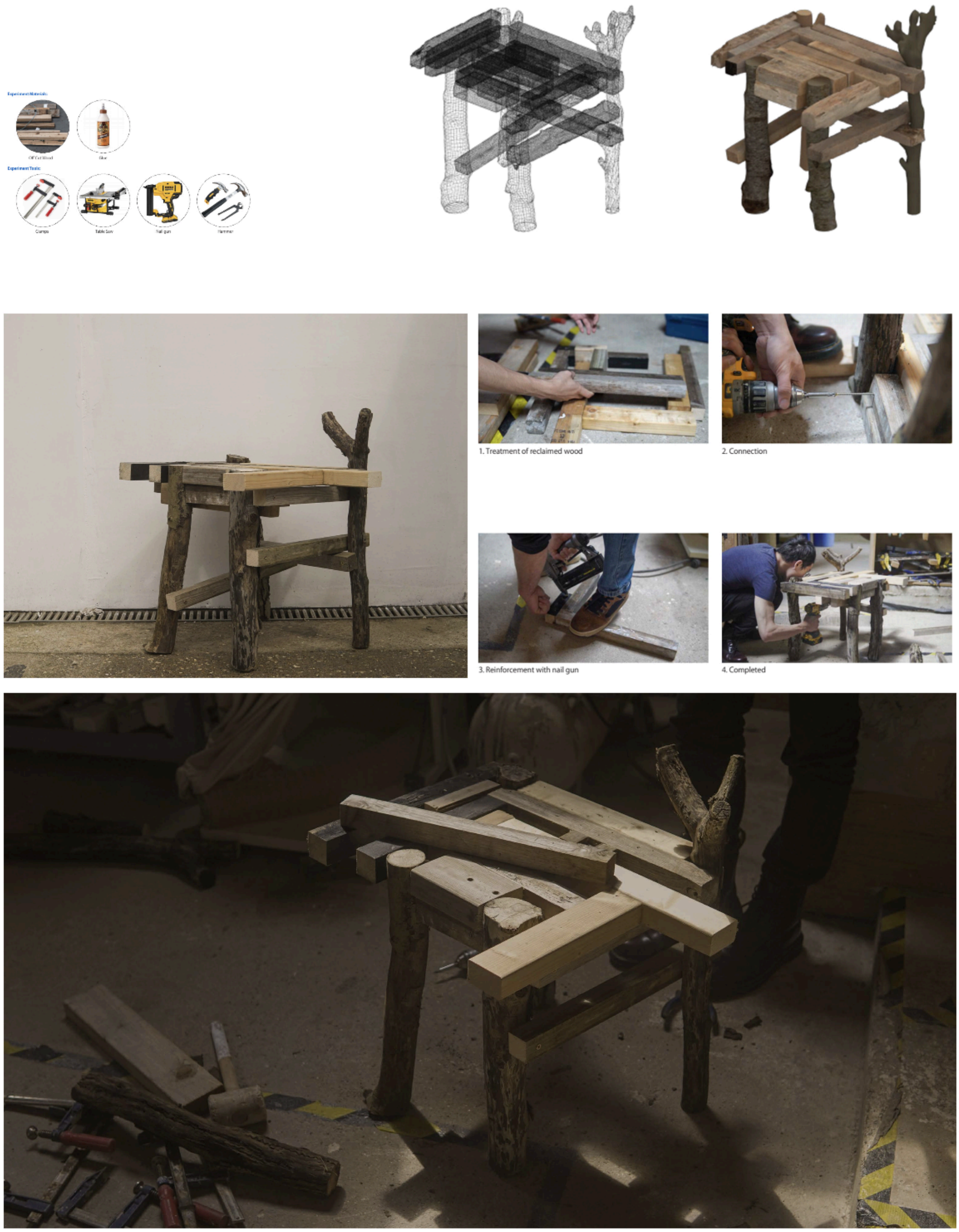

Supplementary Figure 3. Tools and processes for making chairs from irregular wood.

Prototype 02

Melt poly rope joinery 01: Drilled several corresponding holes in the wood.

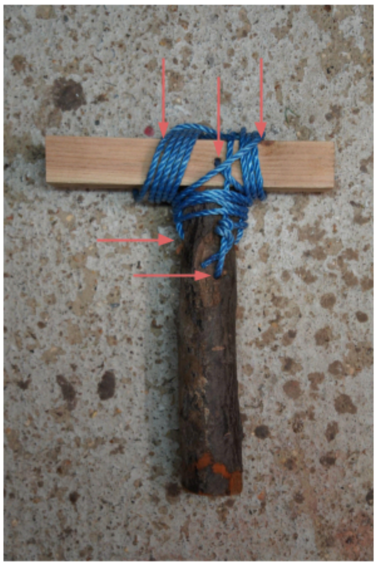

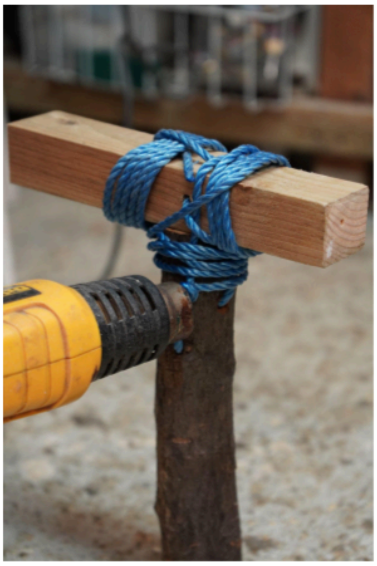

Prototype 02

Melt poly rope joinery 02: Only one hole in the vertical wood.

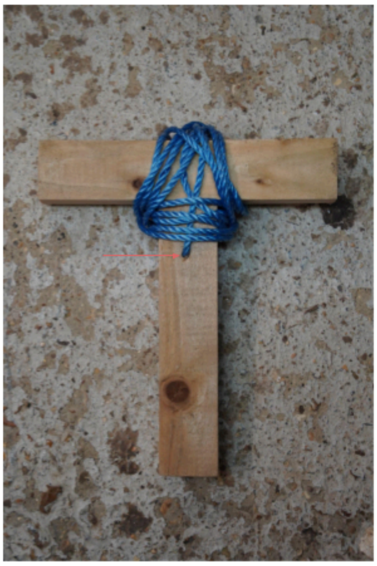

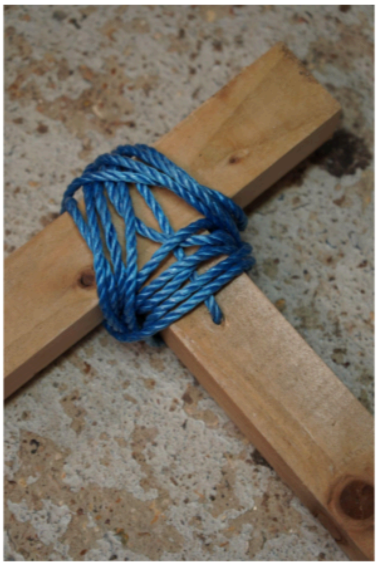

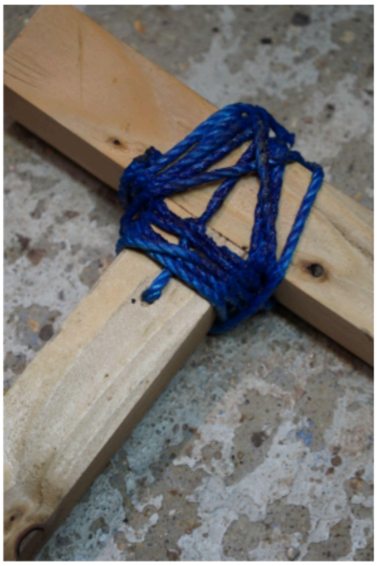

Prototype 02

Melt poly rope joinery 03: Without any hole in the wood.

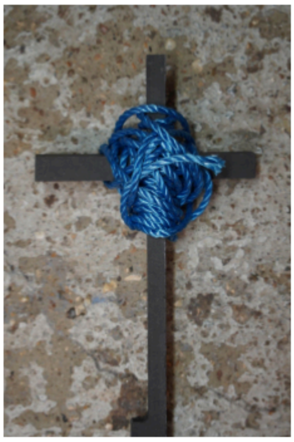

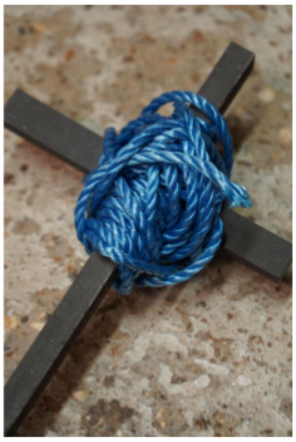

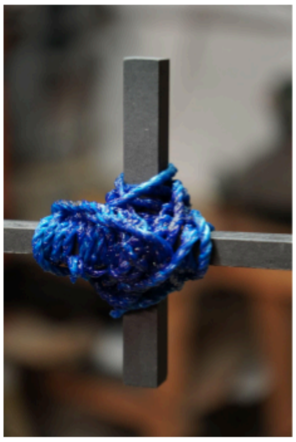

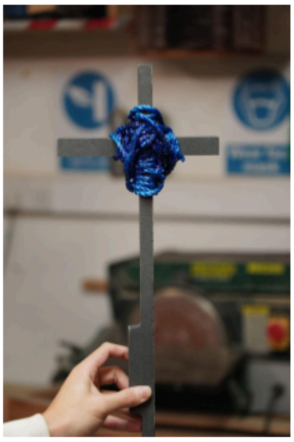

Prototype 02

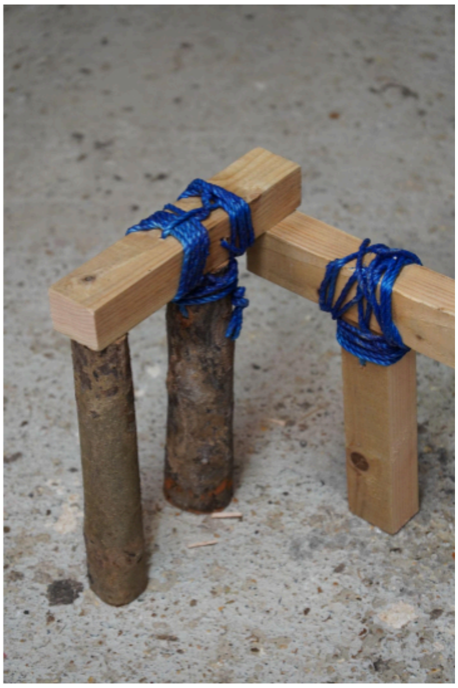

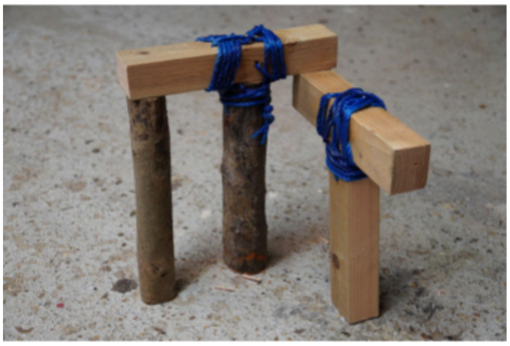

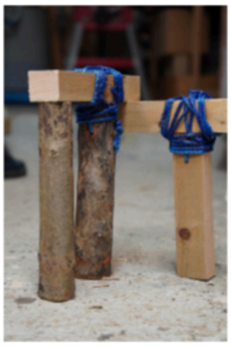

Prototype 01

Shrink plastic joinery 01: Bundle several pieces of off-cut wood together

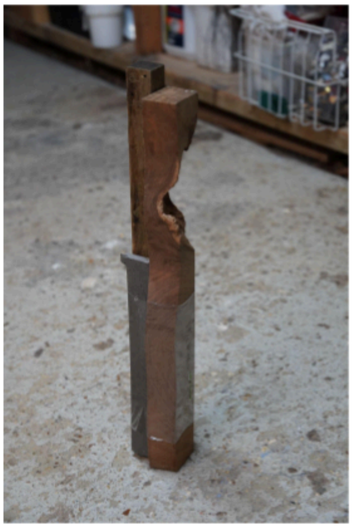
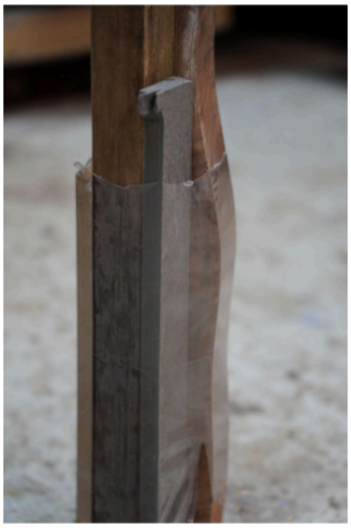
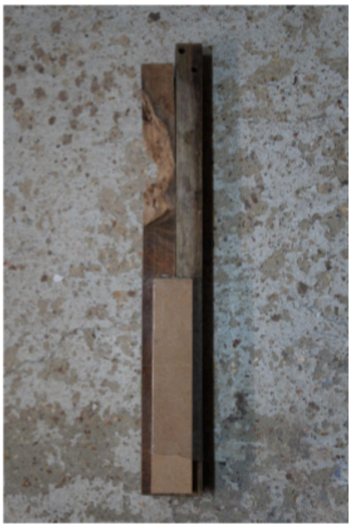

Prototype 01

Shrink plastic joinery 02: Extend and bundle off-cut wood together

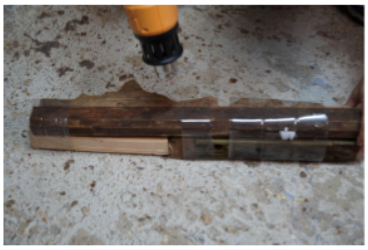
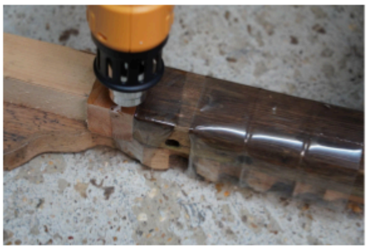
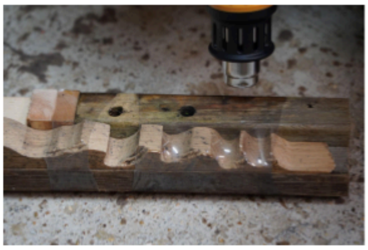

Prototype 01

Shrink plastic joinery 03: Extend and bundle irregular wood together

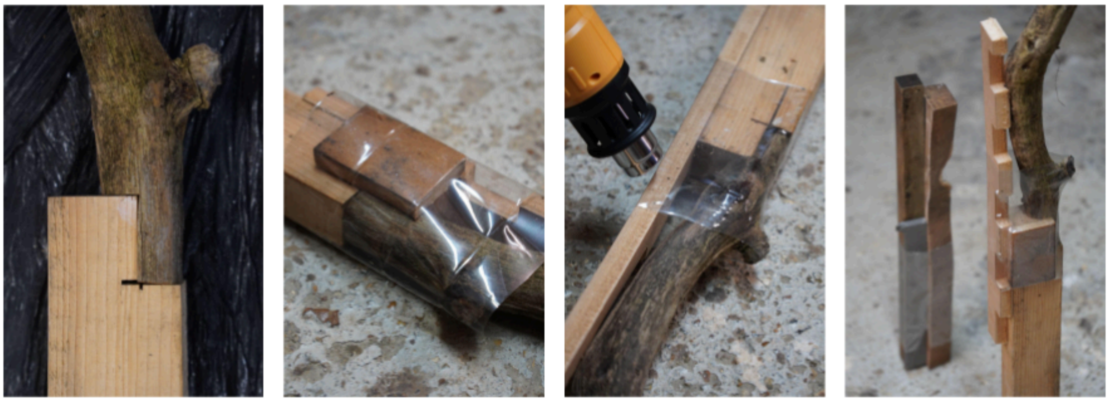

Prototype 01

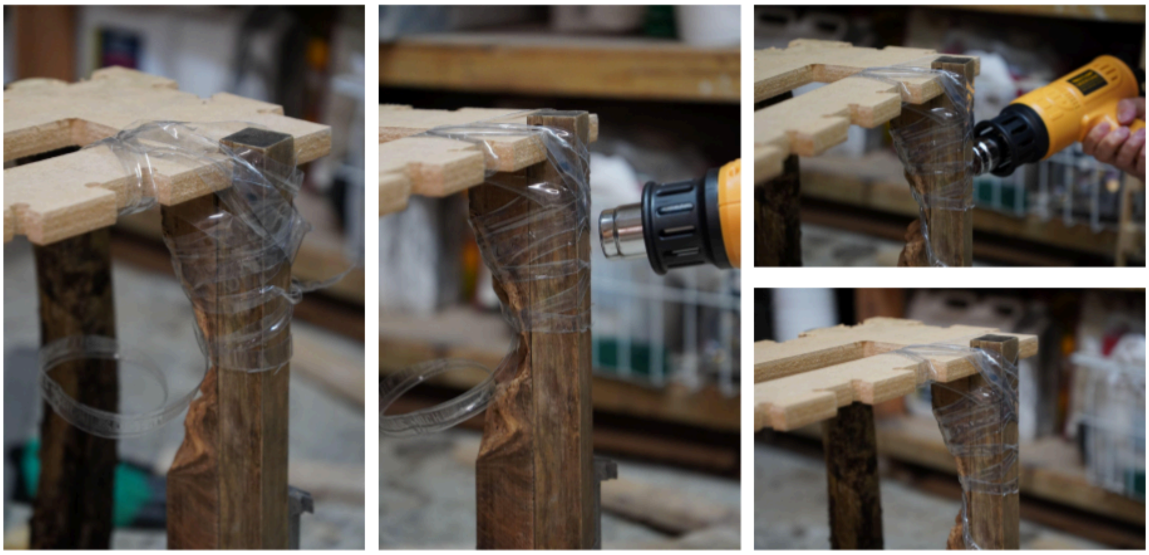

Prototype 01

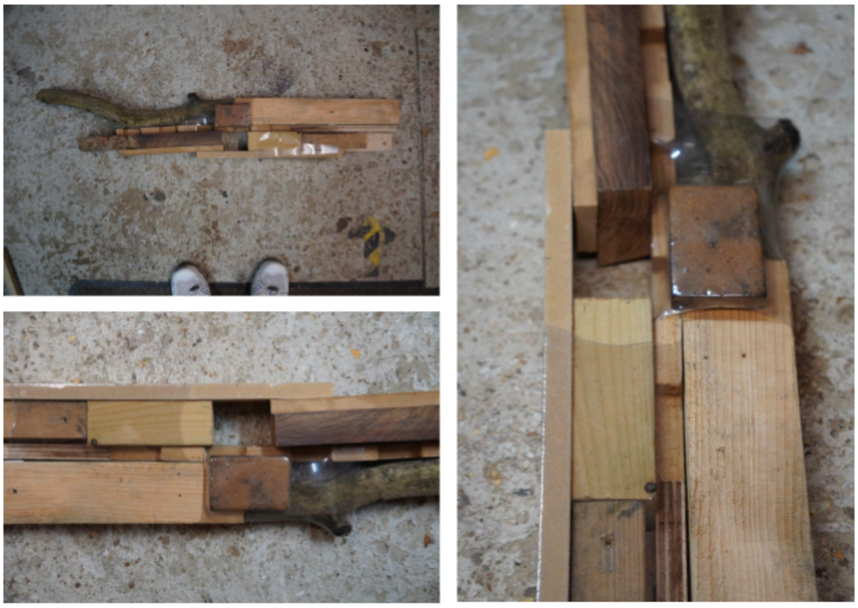

Prototype 01

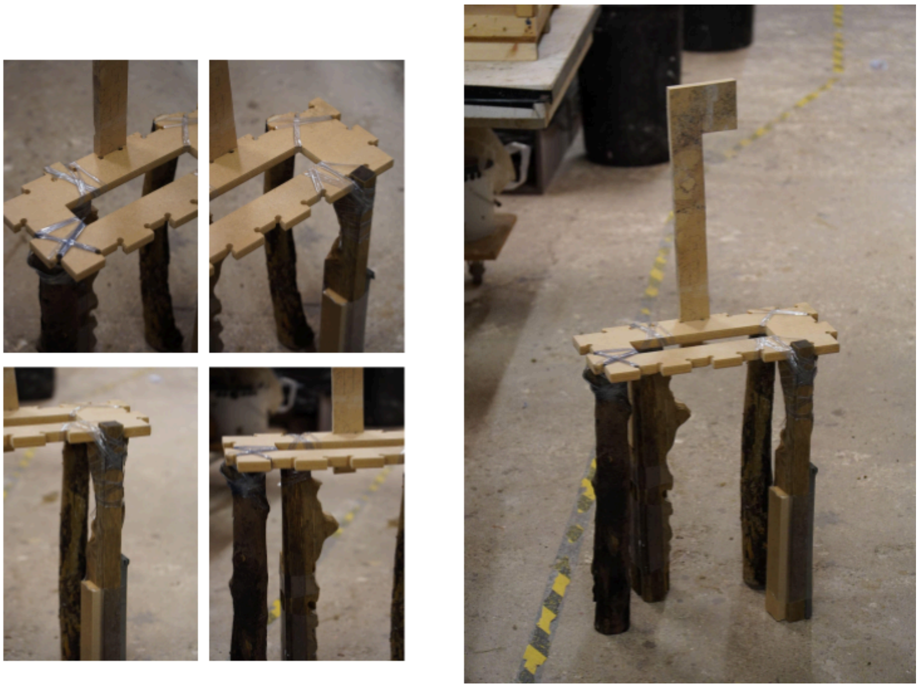

Supplementary Figure 3-12. Fabrication details of irregular wood with plastic heat-moldable joinery

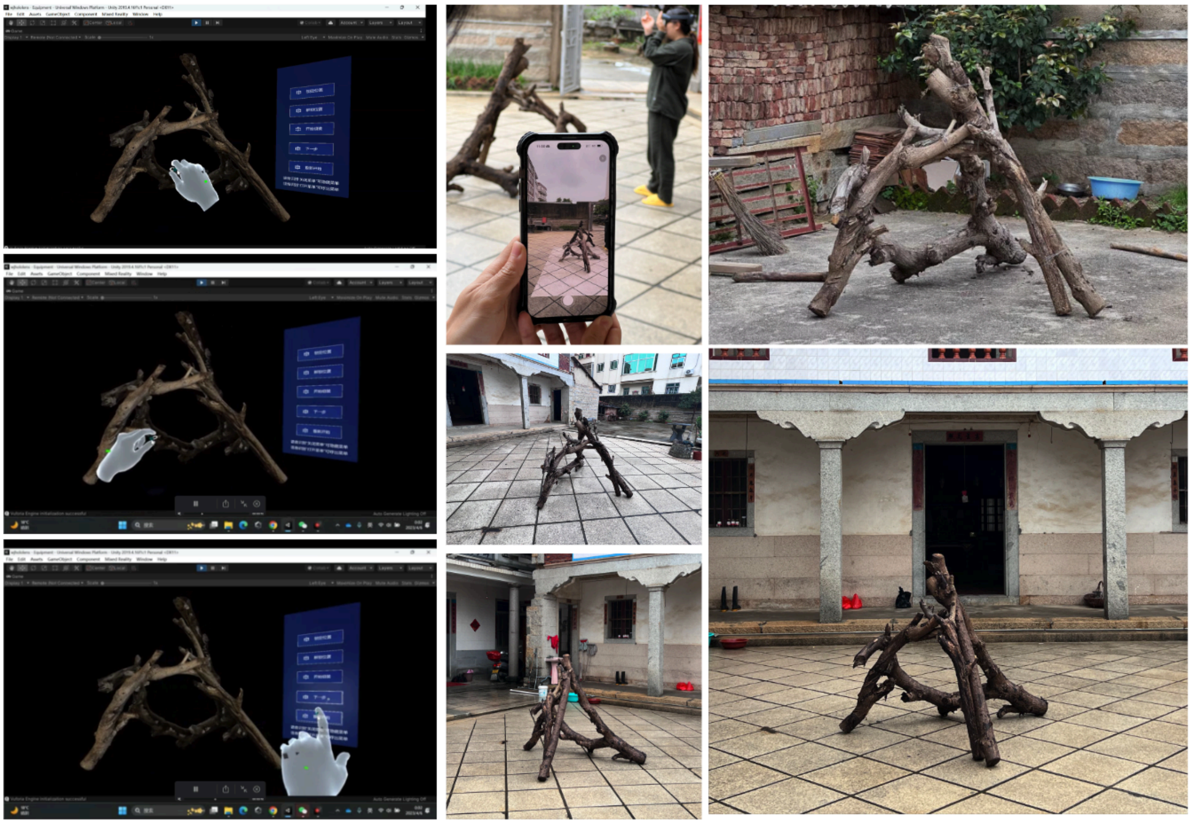

Supplementary Figure 13. First prototype development using an irregular pavilion design and assembly.

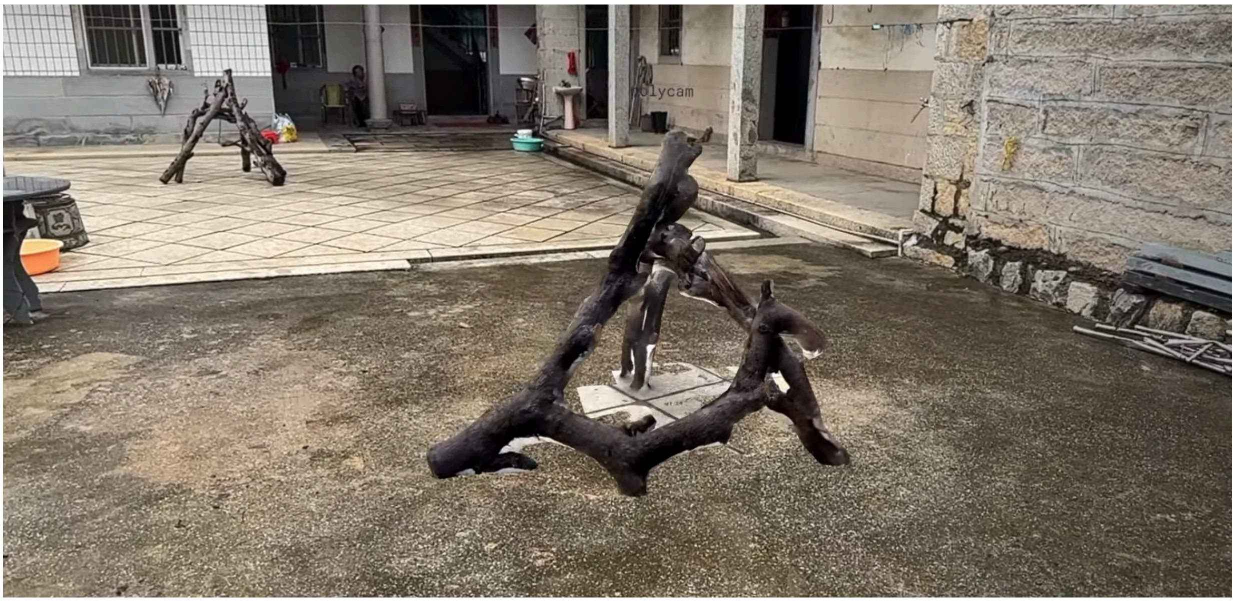

Supplementary Video 1:
https://youtu.be/9P0wJf1PUmI?si=sXvORAksiPqnvCy9

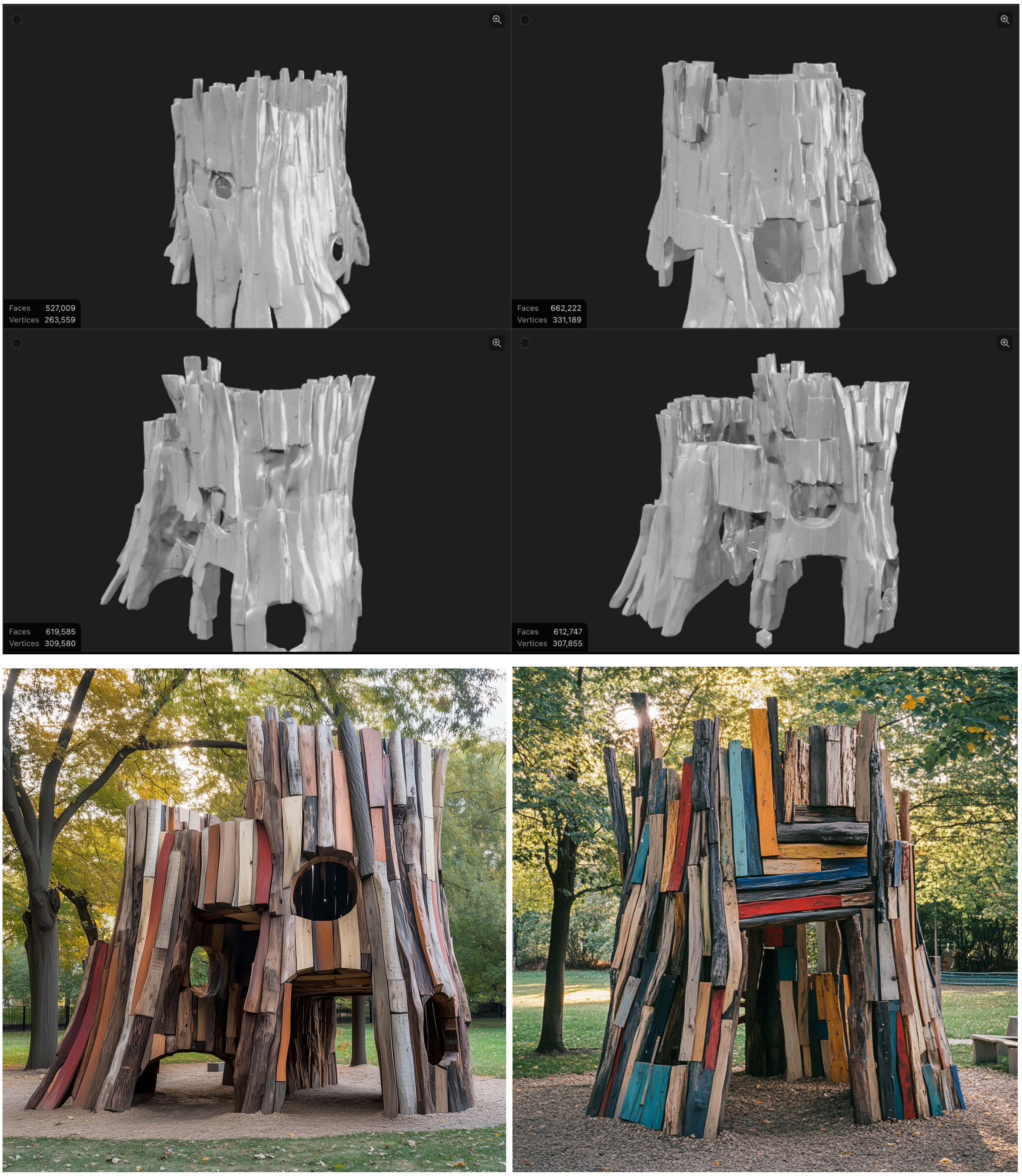

Supplementary Figure 14. AR-assisted visualisation for design decision

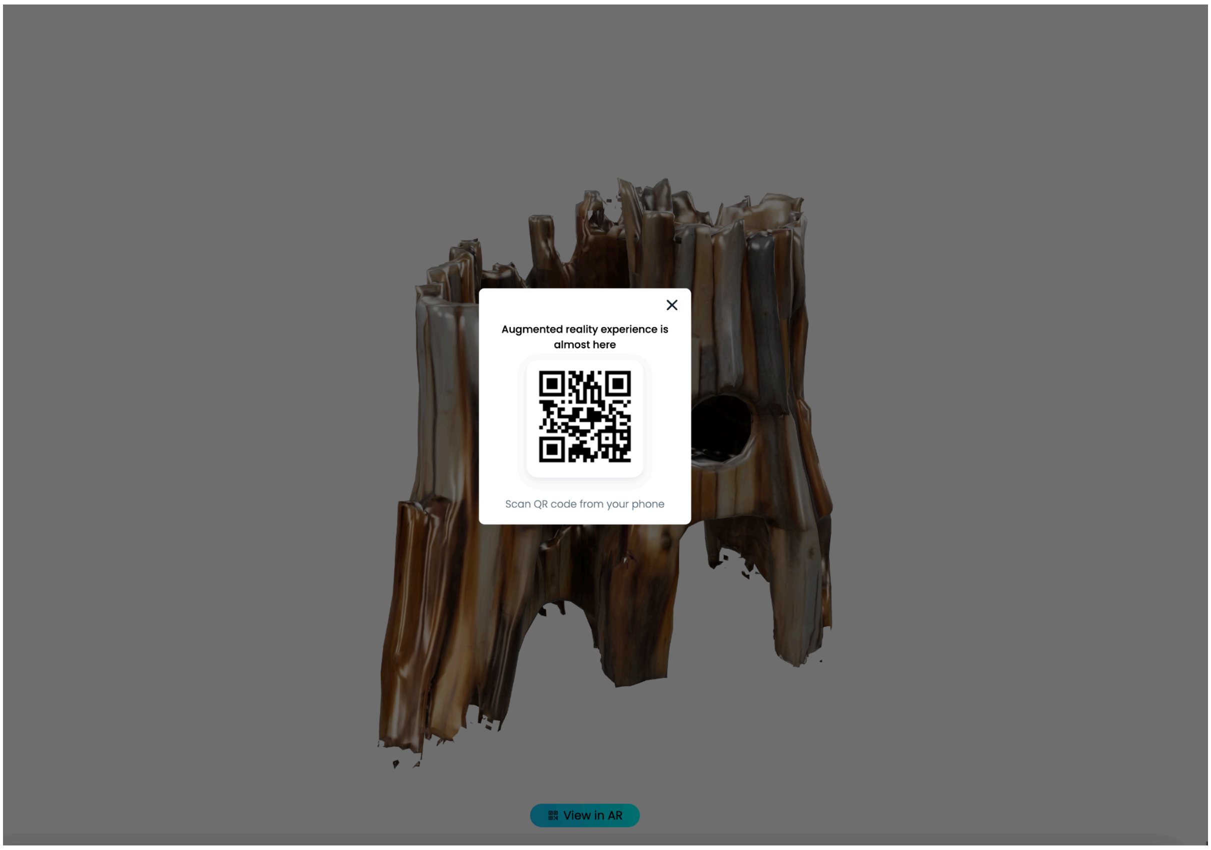

Supplementary Figure 15. QR code for the AR environment. https://app.w3rlds.com/E-tP_bfk

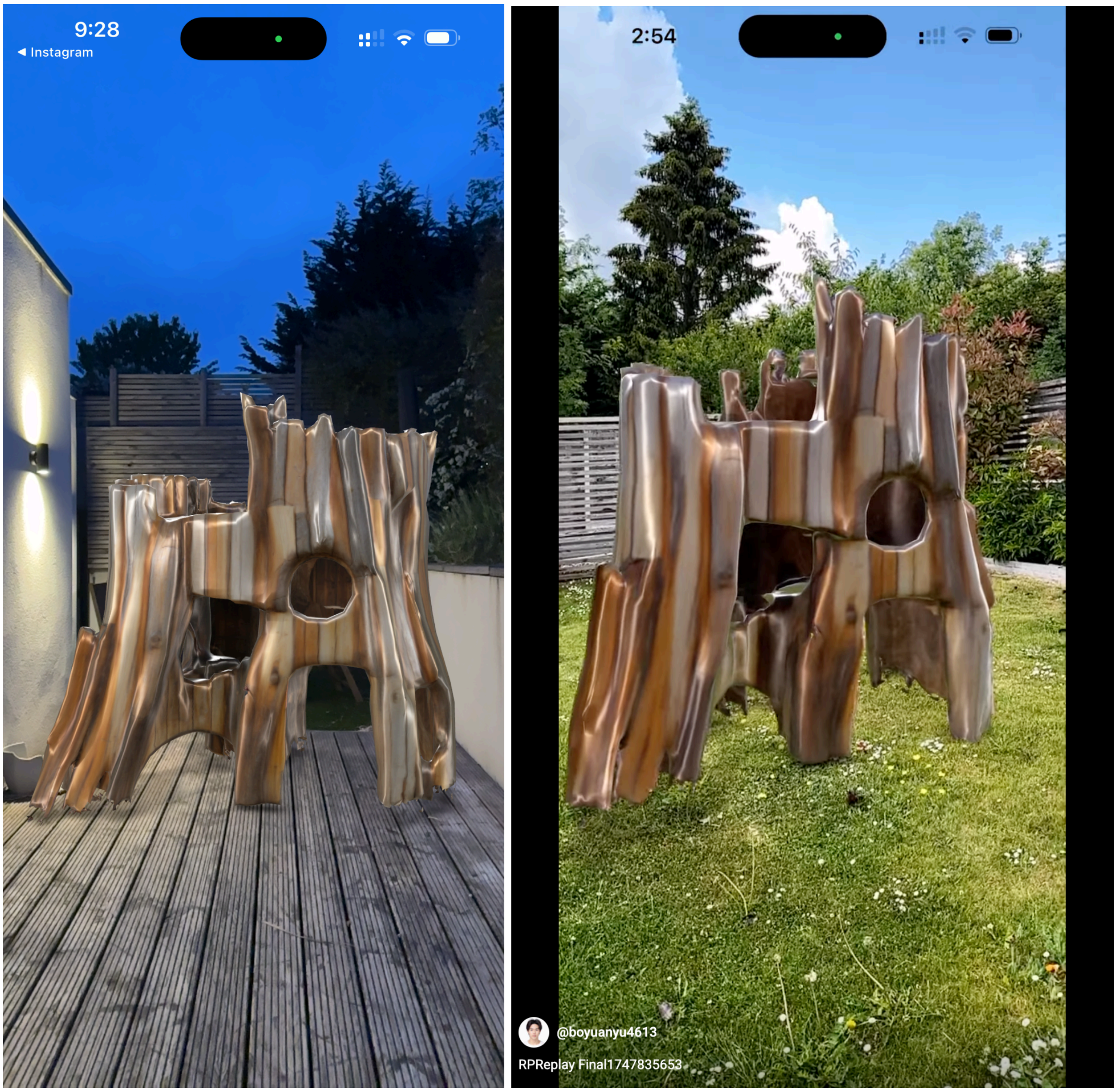

Supplementary Video 2: Architectural proposal in AR environment.
https://youtube.com/shorts/r5bm4xejy1c?si=e5OjZjFTUKY2dBci

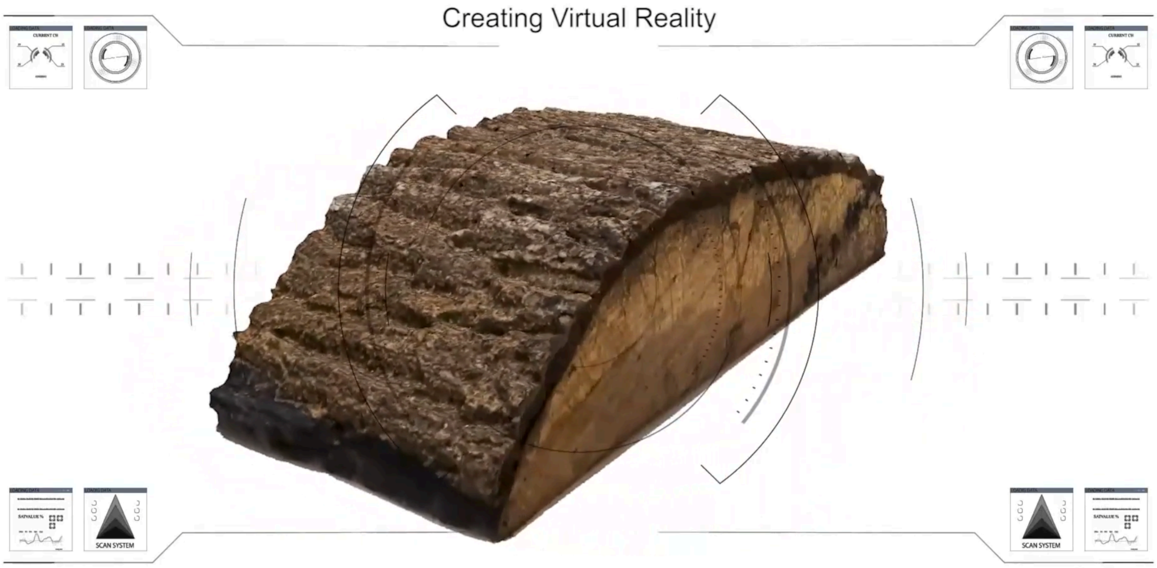

Supplementary Video 3: 3D Scanning Technologies.
https://youtu.be/BeZFmnJoqHM?si=rAYJGW76JNA51I4O
